# The BEN domain protein LIN-14 coordinates neuromuscular positioning during epidermal maturation

**DOI:** 10.1016/j.isci.2024.111577

**Published:** 2024-12-12

**Authors:** Eugene Jennifer Jin, Yingchuan Billy Qi, Andrew D. Chisholm, Yishi Jin

**Affiliations:** 1 Department of Neurobiology, School of Biological Sciences, University of California, San Diego, La Jolla, CA 92093, USA; 2 Department of Cell and Developmental Biology, School of Biological Sciences, University of California, San Diego, La Jolla, CA 92093, USA; 3 Kavli Institute of Brain and Mind, University of California, San Diego, La Jolla, CA 92093, USA; 4 School of Life Science and Technology, ShanghaiTech University, Shanghai 201210, China

**Keywords:** Molecular biology, Neuroscience, Cell biology

## Abstract

Development and function of an organism depend on coordinated inter-tissue interaction. How such interactions are maintained during tissue renewal and reorganization remains poorly understood. Here, we find that *Caenorhabditis elegans* BEN domain transcription factor LIN-14 is required in epidermis for maintaining the position of motor neurons and muscles during developmental tissue reorganization. *lin-14* loss of function *(lf)* mutants display highly penetrant ventral neuromuscular mispositioning. These defects arise post-embryonically during first larval (L1) stage as the maturing epidermis replaces the embryonic ventral epidermis. Tissue-specific and temporally controlled depletion experiments indicate LIN-14 acts within the epidermis for ventral neuromuscular positioning. *lin-14(lf)* mutants show defects in formation of epidermis-muscle attachment complex hemidesmosomes in the maturing ventral epidermis, leading to detachment of muscles and motor neurons as well as movement defects. Our findings reveal a cell non-autonomous role for LIN-14 in coordinating inter-tissue interaction and neuromuscular positioning during epidermal maturation.

## Highlights

• LIN-14 acts in early post-embryonic development to ensure neuromuscular positioning
• LIN-14 functions in epidermis to coordinate neuro-muscle-epidermal reorganization
• LIN-14 regulates formation of hemidesmosomes during epidermal maturation

## Graphical abstract

**Figure:**
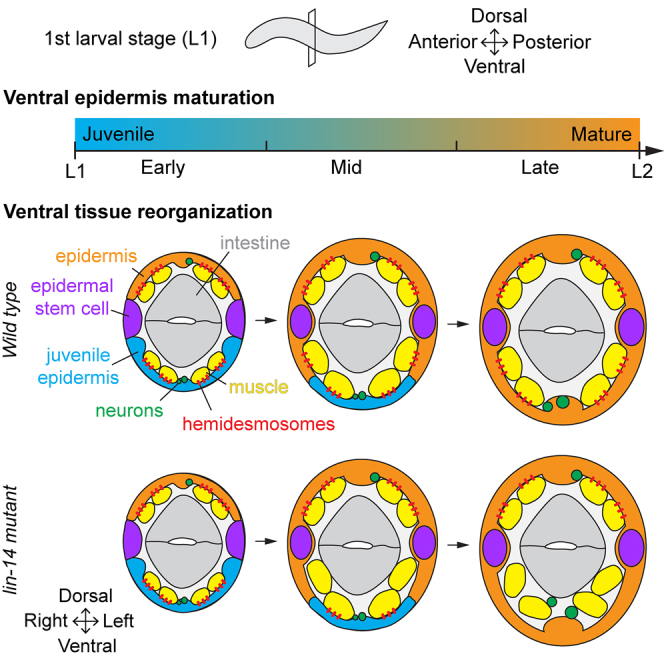

## Introduction

The development of multicellular organisms involves temporal coordination of multiple tissues via systemic and local signals,^1^^,^^2^^,^^3^^,^^4^ as well as inter-tissue interactions involving extracellular matrices (ECMs) and cell-matrix junctions.^5^ Development of skin layers and their associated tissues exemplifies such interactions as it requires coordinated formation of epidermal epithelia, inner muscle or dermal layers, as well as cutaneous innervation. In mammals, postembryonic growth of the epidermis is driven by expansion of self-renewing stem cells whose division patterns are oriented by underlying collagen ECM.^6^ In ecdysozoa, the skin forms the exoskeleton and is repeatedly formed in globally coordinated molts or metamorphoses.^7^^,^^8^ Establishment and maintenance of inter-tissue connections is therefore critical for coordinating tissue organization, patterning and physiological functions (e.g., movement) in multicellular organisms.

In *Caenorhabditis elegans*, connections between epidermis, neurons and muscles are maintained or remodeled during larval growth to allow continued physiological functions (e.g., movement, response to environment) as well as addition of new functions (e.g., reproduction). Post-embryonic skin growth is driven by lateral epidermal stem cells (seam cells).^9^ Post-embryonic maturation of the nervous system involves the remodeling and growth of existing neural processes and addition of post-embryonic neurons.^10^ Growth of the body wall musculature involves enlargement of individual muscle cells as well as addition of post-embryonic muscle cells. Muscle-epidermal connections via epidermal hemidesmosomes (CeHDs: *C. elegans* hemidesmosomes) undergo post-embryonic growth or duplication.^11^ In first larval (L1) stage, the ventral epidermis undergoes maturation,^12^^,^^13^ during which the hyp7 syncytial epidermis makes new CeHDs as it replaces the transient embryonic ventral epidermis (P cells), which rapidly removes CeHDs.^13^ Molecular mechanisms that coordinate such inter-tissue connections during epidermis reorganization remain unknown.

Pioneering studies in *C. elegans* led to the discovery of heterochronic genes^14^ that control timing and progression of cell fates in postembryonic development. As well as causing abnormalities in epidermal or neuroepidermal blast cell lineages, heterochronic genes function in multiple cell types including neurons.^15^^,^^16^^,^^17^ Many components of the heterochronic pathway are conserved throughout metazoans.^18^

*lin-14* is a central regulator of the early larval temporal progression of epidermal and neuronal development.^14^ Loss of function (lf) in *lin-14* results in omission of several developmental events of the L1 stage whereas gain of function (gf) in *lin-14* results in repetition of L1 events in later stages.^14^^,^^19^ *lin-14(lf)* and *lin-14(gf)* mutants both display severe defects in epidermal morphology and in nervous system function. LIN-14 localizes in the nuclei of most cell types in late embryos and L1, and is downregulated in L2 stage.^20^ LIN-14 acts cell autonomously to specify early fates in each tissue. Although initially considered a nematode-specific protein, LIN-14 was recently identified as a member of the conserved BEN (BANP, E5R, and NAC1) domain transcription factor based on protein structure similarity to *Drosophila* Insensitive (Insv) and mouse BEND6.^21^^,^^22^ *Drosophila* Insv functions in peripheral neurogenesis,^23^ as does mammalian BEND6.^24^ Other *Drosophila* BEN proteins act in transcriptional repression and chromatin insulation.^25^ In mammals, BEN domain proteins such as BEND3 maintain pluripotency,^26^ suggesting a common theme of BEN domain proteins acting in early development to repress differentiation programs.

Multiple studies have shown *lin-14* acts in post-mitotic neurons. *lin-14* represses timing of DD neuron synaptic remodeling^27^; it is required in PVT neurons for expression of the ZIG-4 adhesion factor and maintenance of axon fasciculation in the ventral nerve cord (VNC)^28^; it regulates timing of HSN outgrowth^29^; it promotes PLM branch formation^30^; it modulates locomotion through neuropeptide expression^31^; and it limits PVD dendrite arborization.^32^ Functional rescue experiments using cell-type specific expression are broadly consistent with *lin-14* acting cell autonomously in the respective neurons. An exception is the requirement for *lin-14* in touch receptor neuron (TRN) morphology maintenance, which involves *lin-14* function in both TRNs and the surrounding epidermis and muscles.^33^ Notably, most studies of *lin-14* in neural development and function have used partial loss of function *lin-14* mutations that display mild defects in epidermal development.

Here, we show LIN-14 functions in the epidermis to promote neuromuscular organization during developmental epidermis maturation. By analyzing null and strong *lin-14* loss of function mutants, we reveal LIN-14 is critical for stable attachment of ventral muscles and neurons to the ventral epidermis undergoing L1-specific maturation. Auxin-mediated LIN-14 depletion in the epidermis, but not in muscles or neurons, caused ventral muscle detachment and nerve mispositioning. We show epidermal LIN-14 is specifically required for the assembly of epidermal CeHDs in the post-embryonic ventral epidermis that replaces the embryonic epidermis during developmental epidermis maturation. Our observations reveal LIN-14’s role in the coordinated development of epidermal cell-matrix attachments, muscles, and the nervous system.

## Results

### *lin-14* is required for proper positioning of the ventral nerve cord motor neurons

**Figure 1:**
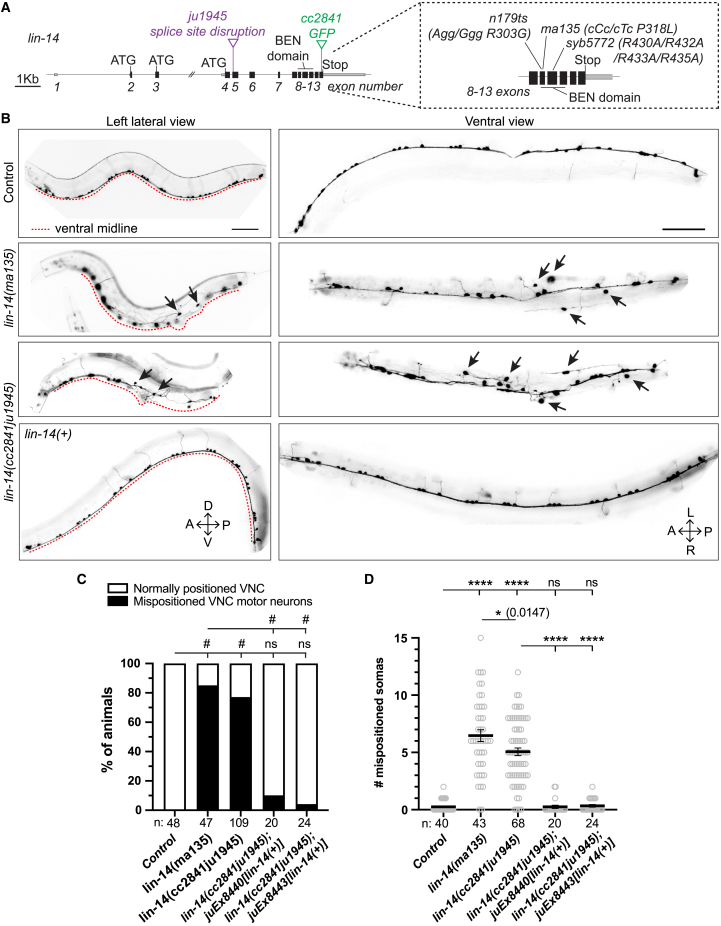
*lin-14* mutants display mispositioned ventral nerve cord (VNC) motor neurons (A) Illustration of *lin-14* alleles. Null alleles *ma135* and *syb5772*, and a partial loss of function allele *n179* have mutations in the BEN domain. *lin-14(cc2841ju1945)* has a 197nt insertion in exon 5 of the *gfp* knock-in allele *lin-14(cc2841)*, resulting in premature stop codons after residue F62. Dashed boxed: enlargement of exons 8–13, including the conserved BEN domain (229–441 aa) encoded in exons 9-11. Scale: 1Kb. (B) Lateral and ventral views of ventral cord cholinergic motor neurons (*juIs14[Pacr-2-gfp]*) in young adults (YAs). Arrows: somas mispositioned from the ventral midline (red dotted line). Scale: 50 μm. (C) Quantification of % of animals displaying mispositioned VNC cholinergic motor neurons in genotypes indicated. Chi-squared test, Marascuilo procedure (#*p* < 0.05, ns *p* > 0.05). (D) Quantification of number of mispositioned cholinergic motor neuron somas, mean ± SEM. One-way ANOVA, Sidak’s multiple comparisons test (ns *p* > 0.05, ∗*p* < 0.05, ∗∗∗∗*p* < 0.0001). See also Figures S1 and S2.

Previous studies have reported a number of *lin-14* loss of function (lf) mutations, ranging from null (*ma135, syb5772*) to partial loss of function (e.g., *n179)* (Figure 1A). *syb5772* contains four missense mutations in the BEN domain.^21^ We identified *ma135* to contain a Pro318Leu missense mutation also in the BEN domain. Both *lin-14* null mutants (herein referred as *lin-14(0)*) exhibit severe locomotor defects, and are unhealthy and sterile,^21^^,^^34^ whereas partial loss of function mutants show mostly normal locomotion and are fertile even at restrictive temperature.^19^

To determine how complete loss of *lin-14* affects motor neurons, we visualized A- and B-type cholinergic and D-type GABAergic motor neurons in *lin-14* null mutants. The *C. elegans* VNC forms two major axon bundles.^35^ Motor neuron somas are linearly positioned within the large bundle of VNC (Figure 1B). In *lin-14(ma135)* adults, somas and axon bundles of cholinergic and GABAergic motor neurons were mispositioned (Figures 1B and S1A). Similar VNC defects were also observed in *lin-14(syb5772)* (Figure S1B)*.* Some somas that were mispositioned >1 soma distance away from the ventral midline often showed misguided and ectopic neurites (Figures S1C and S1D). In contrast, the dorsal nerve cord (DNC), which contains mostly motor neuron axons, appeared largely unaffected (Figure 1B, S1E, and S1F), suggesting that *lin-14* specifically affects VNC motor neuron positioning.

Through genome editing, we generated another loss of function allele, *lin-14(cc2841ju1945),* which contains an insertion of 197 nt in exon 5 that leads to protein out of frame after Phe62 (Figure 1A). LIN-14::GFP(*cc2841*) has GFP in-frame-fused after Leu535 and is fully functional.^36^ Whereas LIN-14::GFP(*cc2841*) was strongly expressed in nuclei of many cell types, *lin-14(cc2841ju1945)* mutants showed undetectable GFP (Figure S2A). *lin-14(cc2841ju1945)* mutant heterozygotes were superficially wild-type in morphology and behavior, whereas homozygous *lin-14(cc2841ju1945)* mutants were dumpy, uncoordinated with reduced locomotion, egg laying defective with ectopic ventral epidermal protrusions, and also male mating defective, similar to *lin-14(0)* (Figures S2B–S2G). *lin-14(cc2841ju1945)* mutants also showed precocious synapse remodeling of GABAergic motor neurons in newly hatched L1s (Figure S2H). VNC motor neuron defects in *lin-14(cc2841ju1945)* were similar to *lin-14(0)* (Figures 1A–1D, S1B). Unlike *lin-14(0),* however, *lin-14(cc2841ju1945)* mutants were semi-fertile, with self progeny broods of up to 15 animals, and could be maintained as homozygotes. By RT-PCR analysis, we detected mRNAs corresponding to exon 7–13 in *lin-14(cc2841ju1945).* It is possible that in *lin-14(cc2841ju1945)* the use of alternative ATG codons 3′ to exon 5 may lead to production of N-terminal truncated proteins at a very low level, as we did not detect visible GFP. Based on these findings, we conclude that *lin-14(cc2841ju1945)* is a strong loss of function mutant.

To further confim that VNC misposition defects are due to loss of function in *lin-14,* we generated *lin-14(+)* transgenes using two independent fosmids. These transgenes fully rescued the gross morphology defects and the VNC motor neuron mispositioning in *lin-14(cc2841ju1945)* mutants (Figures 1C and 1D), showing that LIN-14 is required for proper positioning of motor neurons along the ventral midline. As *lin-14(0)* mutants are sterile, and most visible and VNC defects in *lin-14(cc2841ju1945)* were comparable to *lin-14(0),* we primarily used *lin-14(cc2841ju1945)* in further analysis (for simplicity, designated as *lin-14* mutants).

### VNC motor neuron mispositioning in *lin-14* mutants begin in mid L1

**Figure 2:**
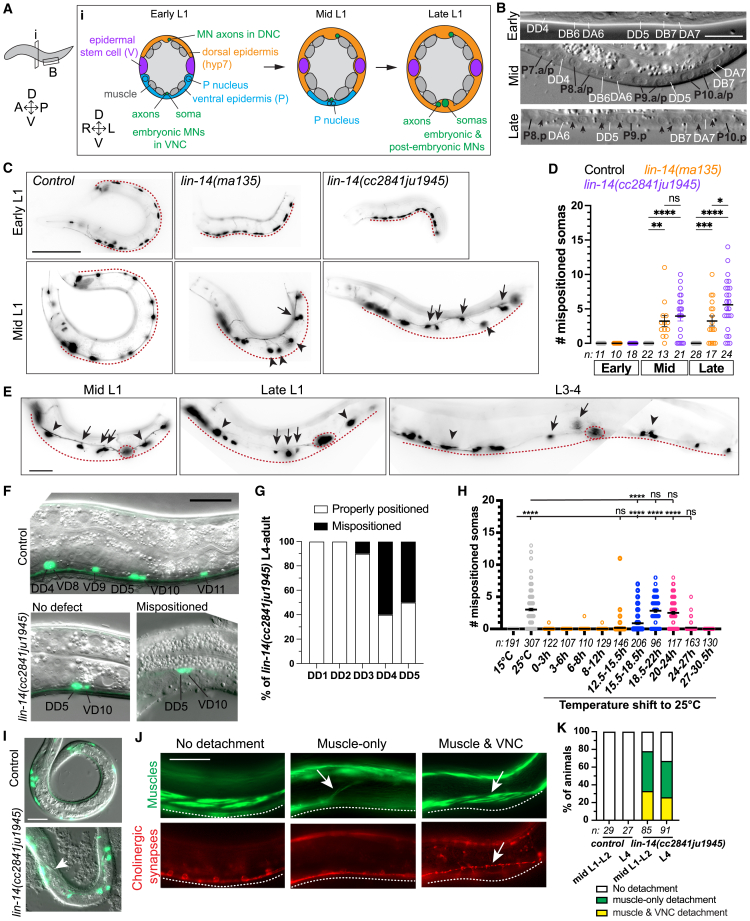
VNC and muscle detachments in *lin-14* mutants begin in mid L1 (A) P cell transition from temporary ventral epidermis in early L1 to blast cells in mid-late L1. MN: motor neurons. VNC: ventral nerve cord (contains MN axons and somas). DNC: dorsal nerve cord (contains MN axons). (B) DIC microscopy images show developmental landmarks used to stage L1s (see STAR Methods for details). Scale: 10 μm. (C) Compound fluorescence images of cholinergic motor neurons in early and mid L1 of control and *lin-14* mutants. Scale: 50 μm. (D) Quantification of mispositioned cholinergic motor neuron somas in L1s, mean ± SEM. One-way ANOVA, Sidak’s multiple comparisons test (ns *p* > 0.05, ∗*p* < 0.05, ∗∗*p* < 0.01, ∗∗∗*p* < 0.001, ∗∗∗∗*p* < 0.0001). (E) Compound fluorescence images of cholinergic motor neurons in the same *lin-14* mutant from mid L1 to L3-4. (C, E) Arrow: severely mispositioned soma. Arrowhead: mildly mispositioned soma. Red dotted circles: properly positioned soma. Red dotted line: ventral midline. Scale: 20 μm. (F) Merged images of DIC and fluorescence showing GABAergic motor neurons (*juIs76[Punc-25-GFP])* in L4 control and *lin-14* mutants. Embryonic (DD) and postembryonic (VD) neurons were identified based on positioning along the VNC. No defect: 6/13 mutants displayed properly positioned DD5 and VD10 on VNC. Mispositioned: 7/13 mutants displayed both DD5 and VD10 mispositioned together. Scale: 20 μm. (G) % of individual embryonic DD neurons mispositioned from the VNC in *lin-14* mutant L4-adults (*n* = 10). *juIs145[Pflp-13-GFP]* was used to label embryonic DD neurons. (H) Quantification of mispositioned VNC cholinergic motor neuron somas after temperature shift of *lin-14(n179ts)* mutant from 15°C to 25°C during embryo development (orange; 100% embryos), hatching period (blue; mixed population of embryos and L1s), or early L1 (pink). Hours (h) on the x axis indicate hours post egg laying. In the early L1 (pink) condition, V and P cells were assessed to determine L1 stages. 20–24 h post egg laying: early L1. Cholinergic motor neuron reporter: *juIs14[Pacr-2-GFP].* Seam cell reporter to determine V cell divisions: *wIs54[scm:gfp].* Mean ± SEM. ANOVA, Dunnett’s multiple comparisons test (ns *p* > 0.05, ∗∗∗∗*p* < 0.0001). (I) Merged images of DIC and fluorescence showing cholinergic motor neuron in mid L1 of control and *lin-14* mutant. Arrow: detached muscle band. Scale: 20 μm. (J) Co-labeling of muscles using UNC-54::GFP*(cc2856*) and cholinergic synapses using UNC-17::mKate2 (*ot907*) in control and *lin-14* mutant mid L1. Arrows: VNC and muscles detached together or separately from ventral midline (white dotted line). Scale: 20 μm. (K) Quantification of % of control and *lin-14* mutant animals displaying VNC and muscle detachment in mid L1-L2 and L4 stages.

VNC motor neurons are born in two phases. The embryonic and early L1 VNC consists of three classes of motor neurons: GABAergic DD and cholinergic DA and DB neurons. From mid L1 to early L2, Pn.a cells divide to generate five additional classes of motor neurons: the GABAergic VD, and the cholinergic VA, VB, VC, and AS neurons.^37^^,^^38^ To determine when the VNC motor neuron mispositioning began in *lin-14* mutants we used developmental landmarks.^37^ Since *lin-14* specifies L1 developmental events, we focused on L1 cell lineage patterns of V and P cells (Figure 2A). In *lin-14* mutant L1s, V cells precociously display L2 lineage patterns whereas P cell development is unaffected.^19^ We therefore used P cell lineage patterns to define early, mid and late L1 stages (Figures 2A and 2B) (see STAR Methods for details). Early L1 *lin-14* mutants displayed normal motor neuron morphology and soma positioning in the ventral midline. Mid L1 *lin-14* mutants displayed mispositioned VNC motor neurons that often appeared as a subset of somas that were mispositioned together with axons from the ventral midline (Figure 2C). These observations suggest the beginning of VNC motor neuron mispositioning in *lin-14* mutants coincides with the timing of P cell maturation from ventral epidermal cells to blast cells.

We noticed that the mean number of mispositioned cholinergic motor neuron somas in *lin-14* mutants remained similar between mid L1 and L4 stages, ranging between 3 and 6.5 (Figures 1D and 2D), suggesting that *lin-14* may selectively affect embryonic motor neuron positioning, or that soma positioning may be restored in later stages. To distinguish between these possibilities, we observed individual *lin-14* mutants from mid L1 to adult. In *lin-14* mutants, neuronal somas that were severely mispositioned (>2 soma widths) from the ventral midline in mid L1 remained mispositioned in later stages (Figure 2E). Using a *Punc-25-GFP* marker to visualize embryonic DD and postembryonic VD neurons, we determined that both DD and VD neurons were mispositioned together (Figure 2F). We also visualized embryonic DD neurons using a *Pflp-13-GFP* reporter and observed that in *lin-14* mutant L4s or adults, DD3-5 neurons were mispositioned, whereas DD1 and DD2 were in normal positions (Figure 2G). In addition, we observed misguided and ectopic neurites of DD neurons beginning in mid L1 near severely mispositioned somas (Figure S1G), suggesting that neurite defects might be a response to severe VNC motor neuron mispositioning. We conclude that LIN-14 is required for positioning of the embryonically generated VNC motor neurons at the ventral midline in mid L1, and that placement of postembryonic motor neurons is likely guided by the position of embryonic VNC neurons. The total number of displaced somas did not increase from mid L1 to L4, possibly due to partial restoration of mildly mispositioned somas. These results point to events in the mid L1 stage as critical for positioning of the VNC motor neurons.

LIN-14 is most highly expressed from late embryo to L1^20^^,^^36^, suggesting that proper positioning of the VNC motor neurons may require LIN-14 function in embryo, early L1, or both. To determine when *lin-14* function is required for VNC motor neuron positioning, we used *lin-14(n179ts)* to temporally restrict *lin-14* function during embryo development, during hatching or in early L1 after hatching (Figure 2H). *lin-14(n179ts)* mutants displayed normal VNCs at the permissive temperature (15°C), and 45% VNC defects at the restrictive temperature (25°C) (Figures S1H and S1I). Restricting *lin-14* function in early L1 (20–24 h post egg laying) or during hatching period (18.5–22 h post egg laying) resulted in similar numbers of mispositioned motor neurons. However, restricting *lin-14* function during embryo development, during early hatching period (15.5–18.5 h post egg laying), or from mid L1 (24 h post egg laying) did not cause significant mispositioning. Thus, we conclude that proper positioning of the VNC motor neurons in mid L1 requires post-embryonic LIN-14 function in early L1.

### Ventral muscle detachment in *lin-14* mutants also begins in mid L1

Body wall muscles are adjacent to the VNC and motor neurons form synapses with the muscles. We next addressed whether positioning of ventral muscles was also affected in *lin-14* mutants. Body wall muscles form two ventral quadrants flanking the VNC and two dorsal quadrants flanking the DNC via attachments to ventral and dorsal epidermis, respectively. Using DIC microscopy, we observed that compared to control animals, mid-late L1s of *lin-14* mutants frequently displayed detached ventral muscle bands (Figure 2I). To determine if the VNC motor neuron mispositioning and ventral muscle detachment in *lin-14* mutants occurred together or independently, we visualized body wall muscles (UNC-54::GFP) and VNC cholinergic synapses (UNC-17::mKate). In *lin-14(cc2841ju1945)* mutants, co-detachment of ventral muscles and the VNC was observed in 26–33% of mutants (Figures 2J and 2K). 41–46% of mutants displayed muscle detachment but normal positioning of the VNC cholinergic synapses; we did not observe mispositioning of VNC cholinergic synapses without muscle detachment. Moreover, the percentage of mutants displaying detachments did not increase from mid L1 to L4 (Figure 2K), suggesting that the ventral muscle-nerve detachments in *lin-14* mutant occur during mid L1 and do not progress in later stages. Together, we conclude that loss of *lin-14* causes mid L1-specific mispositioning of the embryonically born VNC motor neurons, as well as detachment of ventral body wall muscles from ventral epidermis.

### LIN-14 functions in the epidermis to position ventral nerves and muscles

LIN-14 is expressed in most tissues, including the nervous system, muscles and epidermis.^20^^,^^36^ To determine in which tissue(s) LIN-14 function is required for proper positioning of the VNC motor neurons and ventral muscle attachment to epidermis, we used the auxin-mediated conditional protein depletion system. We tagged endogenous LIN-14 at the C-terminus with AID∗::mScarlet-I (assigned *ju1965*) (Figure S3), and then introduced transgenes expressing TIR1 in neurons, body wall muscles, epidermis or intestine.^39^ We confirmed efficient auxin-mediated tissue specific depletion of LIN-14 based on loss of LIN-14::mScarlet-I expression (Figures S4A and S4B). Epidermal depletion of LIN-14 caused vulval protrusions, egg laying defects and small body size, consistent with previous findings that the *lin-14* regulator *lin-4* functions autonomously in the epidermis^40^ (Figures S5A and S5B). Neuronal depletion of LIN-14 caused precocious developmental synapse remodeling of GABAergic motor neurons^27^ (Figure S5C), as well as defasciculation of axon bundles along the VNC (Figures S5D–S5F and 3B), consistent with previous findings.^28^ LIN-14 depletion in the muscle or intestine did not cause any gross morphology or motor neuron defects. Thus, tissue specific depletion experiments supported previous conclusions of *lin-14* cell autonomous functions in epidermis and neurons.

**Figure 3:**
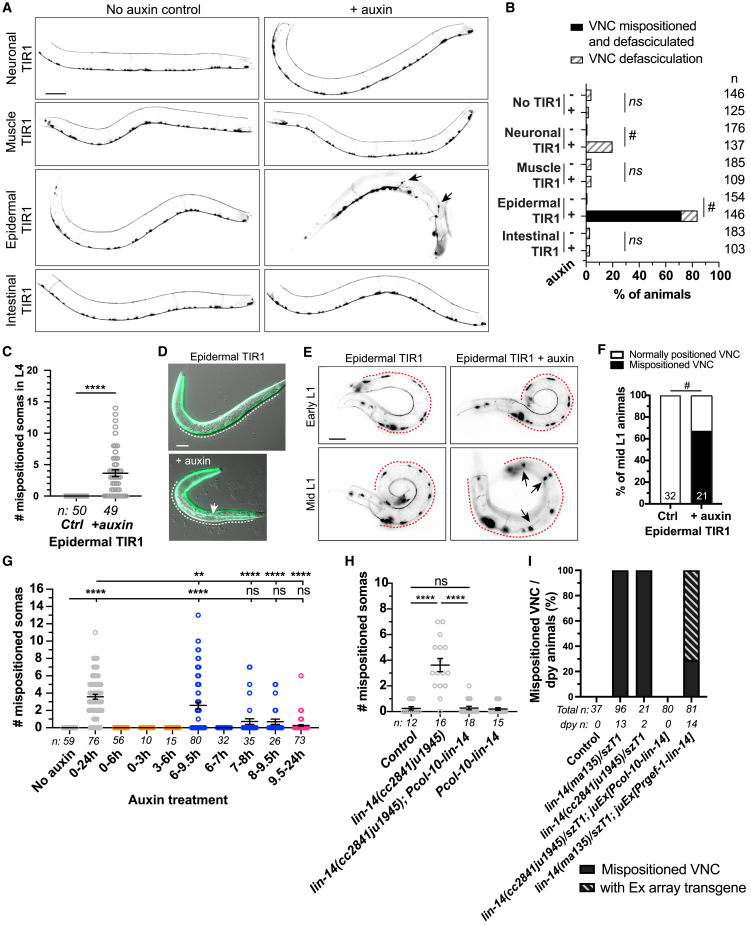
Epidermal LIN-14 is required for VNC and muscle attachment (A and E) Compound fluorescence images of cholinergic motor neurons (*juIs14[Pacr-2-GFP])* in control and auxin-induced tissue specific LIN-14 depletion in L4-YA (A) and early, mid L1 (E). *Pcol-10-*driven epidermal TIR1 was used unless specified. Arrows: mispositioned somas from ventral midline (red dotted line in (E)). Scale: 50 μm (A), 20 μm (E). (B and F) Quantification of % of L4-YA animals (B) or mid L1 animals (F) displaying mispositioned VNC somas or defasciculated VNC after tissue specific LIN-14 depletion. Chi-squared test followed by Marascuilo procedure were performed separately for “VNC mispositioning and defasciculated” and “VNC defasciculation” phenotypes (#*p* < 0.05, ns *p* > 0.05). (C and H) Quantification of mispositioned cholinergic motor neuron somas in L4s. Mean ± SEM. Unpaired t-test (∗∗∗∗*p* < 0.0001) (C); one-way ANOVA, Sidak’s multiple comparisons test (ns *p* > 0.05, ∗∗∗∗*p* < 0.0001) (H). (D) Merged images of DIC and fluorescence showing body wall muscles (UNC-54::GFP*(cc2856)*) in L2 in control and epidermal LIN-14 depletion. Arrow: ventral muscle detached from ventral midline (white dotted line). Scale: 20 μm. (G) Quantification of VNC cholinergic motor neuron soma mispositioning after transient epidermal LIN-14 depletion, shown as mean ± SEM. Auxin treatment: hours (h) after post egg laying. Color coding is same as in Figure 2F. One-way ANOVA, Dunnett’s multiple comparisons test (ns *p* > 0.05, ∗∗*p* < 0.01, ∗∗∗∗*p* < 0.0001). (I) Quantification of % of Dpy animals showing mispositioned VNC motor neurons. Total number of animals scored, and number of dpy animals are indicated per genotype. See also Figures S3–S5.

We observed that LIN-14 depletion in epidermis (*Pcol-10-TIR1*) resulted in mispositioning of the VNC motor neurons (Figures 3A–3C) and detachment of ventral muscles (Figure 3D), both beginning in mid L1 (Figures 3D–3F). We next tested temporal depletion of LIN-14 following a pulse of auxin treatment. We observed significant depletion of LIN-14 in neurons or epidermis after 30 min and in muscles about 100 min on auxin treatment (Figures S4C and S4D). Animals after a pulse of auxin treatment during hatching, but not in embryos or after hatching is complete, showed mispositioned VNC motor neurons (Figure 3G). This analysis suggests that epidermal LIN-14 function is required in early L1 for VNC and muscle positioning, consistent with the temperature shift results using *lin-14(n179ts)* (Figure 2H). Similar to *lin-14* mutants, epidermal depletion of LIN-14 also resulted in misguided axons and reduced locomotion (Figures S5G and S5H), suggesting that detached muscles and nerves likely contribute to movement defects. We also used another epidermal TIR1 transgene driven by a *Pdpy-7* epidermis promoter, which resulted in partial depletion of LIN-14 in the lateral epidermis (Figures S4A and S4B). We observed reduced frequency of animals displaying small body size and ventral protrusions (100% of *Pcol-10*-driven LIN-14 depletion animals vs. 20–50% of *Pdpy-7-*driven LIN-14 depletion animals), and no VNC mispositioning (Figure S4E). These results are consistent with the analyses of *lin-14* mutant alleles, supporting that VNC and muscle detachments occur as the result of severe loss of *lin-14* function. Conversely, we asked if *lin-14* expression in epidermis could rescue the ventral detachments observed in *lin-14* mutants. Epidermal expression of *lin-14::GFP* using *Pcol-10* rescued the gross morphology phenotypes and the VNC motor neuron positioning defects of *lin-14* mutants (Figures 3H and 3I). In contrast, expressing *lin-14* in neurons using *Prgef-1* promoter did not rescue gross body morphology or VNC positioning phenotypes (Figure 3I). We conclude that epidermal LIN-14 is necessary for the ventral attachments of nerves and muscles.

### Epidermal LIN-14 is required for proper localization of ventral hemidesmosomes

**Figure 4:**
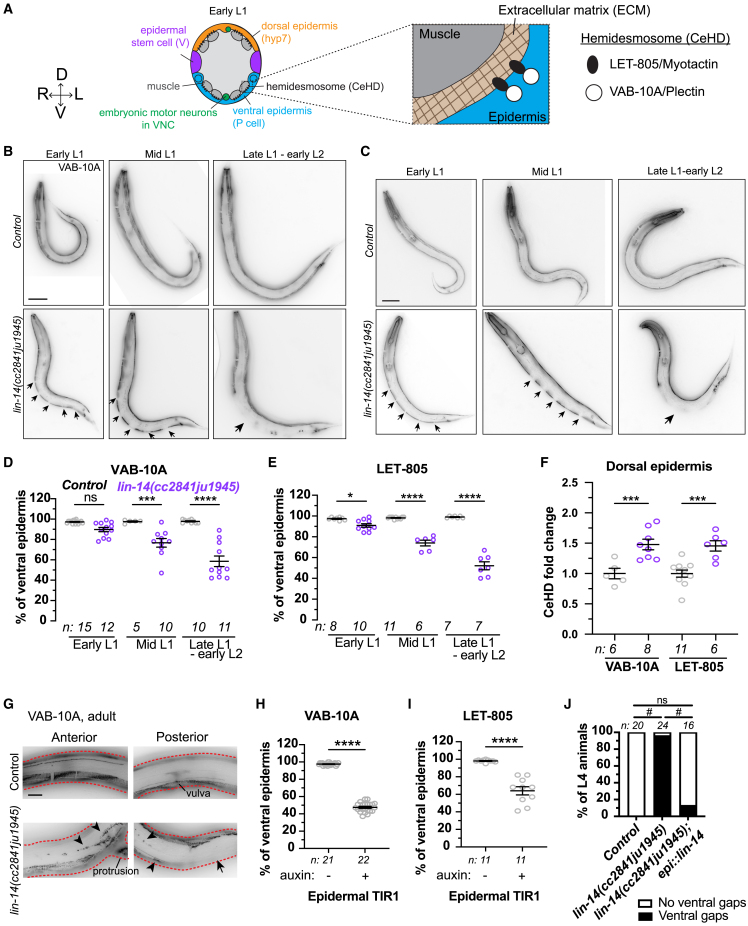
Epidermal LIN-14 depletion causes progressive loss of ventral hemidesmosomes (CeHDs) in L1 (A) Illustration of muscle-epidermis attachments: *C*. *elegans*hemidesmosome (CeHD) components VAB-10A and LET-805 in the epidermis.^45^ (B, C, and G) VAB-10A::GFP (B, G) and LET-805::GFP (C) expression in L1-L2 (B-C) and adult (G) control and *lin-14* mutants. (G) Compound fluorescence images focusing on ventral VAB-10A::GFP expression. Red dashed lines: dorsal (upper) and ventral (lower) boundaries. Arrow: ventral gap. Arrowhead: irregular and disconnected CeHD array. Scale: 20 μm. (D, E, H, and I) Quantification of % of ventral epidermis expressing VAB-10A (D, H) or LET-805 (E, I) in L1 (D-E) or L2 (H-I) animals. Mean ± SEM. One-way ANOVA followed by Sidak’s multiple comparisons test (D-E), and unpaired t-test (H-I) (ns *p* > 0.05, ∗*p* < 0.05, ∗∗∗*p* < 0.001, ∗∗∗∗*p* < 0.0001). (F) Normalized VAB-10A::GFP and LET-805::GFP expression levels in dorsal epidermis (hyp7) in mid L1 control and *lin-14* mutants, indicated by mean ± SEM. One-way ANOVA, Sidak’s multiple comparisons test (∗∗∗*p* < 0.001). (J) Quantification of % of L4 animals displaying ventral VAB-10A gaps. Chi-squared test, Marascuilo procedure (#*p* < 0.05, ns *p* > 0.05).

Attachment of body wall muscles to epidermis is mediated by cell adhesion proteins in the epidermis and extracellular matrix (ECM) between the tissues (Figure 4A), and mutants lacking these attachment components display detached muscles and nerves.^41^^,^^42^^,^^43^ We therefore investigated whether epidermal cell adhesion proteins known as hemidesmosomes (CeHDs: *C. elegans* hemidesmosomes) might be affected by loss of LIN-14. VAB-10A/Plectin^44^ and LET-805/Myotactin^43^ normally localize to regular repeating stripes in the dorsal and ventral epidermis adjacent to body wall muscles throughout embryonic development to adults (Figures 4B and 4C). Both genes were reported to be candidate transcriptional targets of LIN-14.^31^ In *lin-14* mutants, the ventral CeHD stripe arrays contained 5 regular gaps that progressively increased in size from early L1 (∼1.5 μm), mid L1 (∼6.5 μm) to early L2 (Figures 4B–4E). In contrast, dorsal CeHD stripe arrays were evenly distributed from head to tail throughout L1, but with ∼1.5-fold increased expression levels in *lin-14* mutants compared to control (Figure 4F), suggesting that LIN-14 may differentially affect CeHDs in ventral and dorsal epidermis. *lin-14* mutant adults displayed discontinuous and irregular ventral CeHD stripes with large gaps (Figure 4G), suggesting that early defects in ventral localization of CeHDs are irreversible. Moreover, LIN-14 depletion in epidermis (P*col-10*) resulted in loss of ventral CeHDs (Figures 4H and 4I), and epidermal expression of *lin-14::GFP* rescued loss of ventral CeHDs in *lin-14* mutants (Figure 4J). We conclude that epidermal LIN-14 is required for proper localization of ventral, but not dorsal, CeHDs during L1 development.

### LIN-14 is required for CeHD assembly in the maturing epidermis

**Figure 5:**
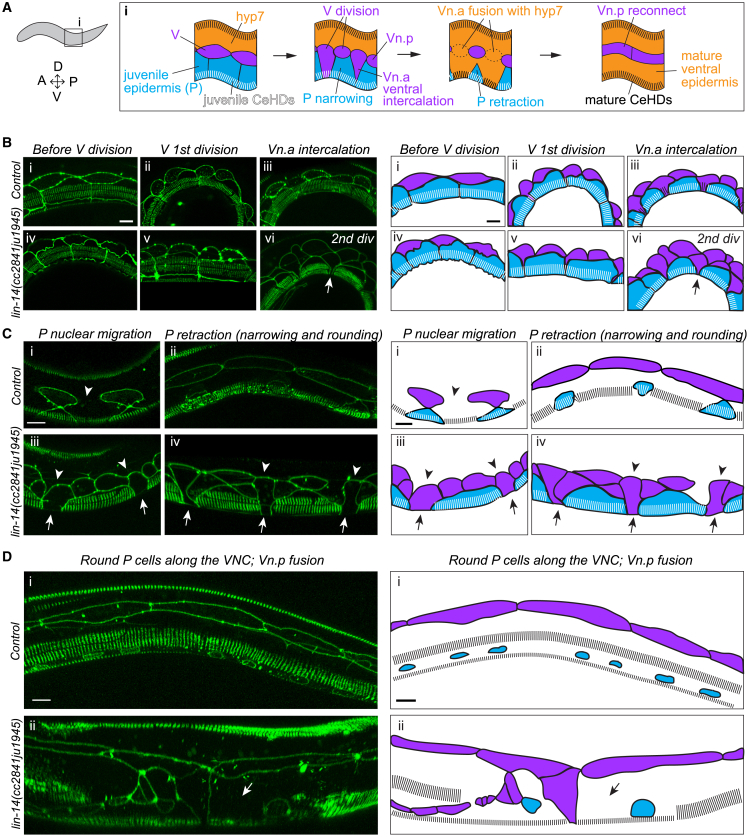
Loss of *lin-14* causes loss of post-embryonic CeHD assembly in maturing ventral epidermis (A) Illustration of lateral view (i) of epidermal tissue reorganization and CeHD assembly/disassembly during ventral epidermis maturation in L1. Dorsal epidermis remains composed of hyp7 throughout the L1. (B–D) Confocal images showing VAB-10A::GFP and epidermal cell junctions (AJM-1::GFP) before (B), during (C), and post (D) ventral epidermis reorganization. Scale: 5 μm. Arrow: ventral gap lacking VAB-10A::GFP. Arrowhead: epidermal cell boundary between Vn.a/Vn.xx and dorsal hyp7 in (C).

To maintain epidermis-muscle connections as the maturing epidermis (hyp7 syncytium) expands and replaces embryonic ventral epidermis (P cells) in L1,^12^ new CeHDs assemble in the maturing ventral epidermis while the P cell CeHDs disassemble^13^ (Figure 5A). To determine if the gaps observed in ventral CeHDs in *lin-14* mutants were in the ventrally retracting juvenile epidermis or in the maturing epidermis, or both, we analyzed VAB-10A localization in P, V, and hyp7 epidermal cells labeled with epithelial adherens junction marker AJM-1::GFP. In control L1s, VAB-10A stripe arrays were present in P cells before and during retraction as the cells narrowed and rounded in shape (Figures 5B and 5C), but not post-retraction after P cells divided (Figure 5Di). VAB-10A stripe arrays also localized to the maturing ventral epidermis throughout ventral intercalation and expansion (Figures 5B and 5C). In *lin-14* mutant L1s, V cells precociously undergo L2-like cell divisions,^14^^,^^19^ resulting in the generation of mature ventral epidermis by V cell granddaughters (Vn.xx) as opposed to anterior daughters (Vn.a) (Figure 5Biii, vi). In *lin-14* mutants, VAB-10A stripe arrays were also present in P cells before and during retraction, and were absent post-retraction (Figures 5B–5D). However, VAB-10A was not detectable in the Vn.xx-derived ventral epidermis (Figure 5Ciii-iv). The Vn.xx descendants without VAB-10A stripe arrays intercalated ventrally between P cells and expanded to replace the retracting P cells, resulting in progressive loss of ventral VAB-10A (Figures 5C and 5D). Our observations suggest that LIN-14 is required specifically for CeHD assembly in the maturing ventral epidermis, whereas CeHD assembly and disassembly in P cells are independent of LIN-14.

We also observed delayed V.xx-hyp7 fusion and P cell retraction during ventral epidermis maturation in *lin-14* mutants. Wild type Vn.a cells fuse with hyp7 after they ventrally intercalate and as they expand to replace P cells (Figure 5Ci). In *lin-14* mutants, however, most Vn.xx descendants maintained dorsal boundaries after ventral intercalation and expansion (Figure 5Ciii-iv), suggesting their fusion with hyp7 is delayed. *lin-14* mutant P cells also appeared wider than in control after ventral intercalation of Vn.xx (Figure 5Ciii-iv), suggesting that the timing of P cell narrowing and rounding may also be delayed, although the P cells were retracted fully to the VNC by L2 (Figure 5Dii). We conclude that LIN-14 is required for the normal reorganization of the ventral epidermis, including formation of new ventral CeHDs that maintain proper attachment and positioning of ventral muscles and nerves.

## Discussion

### The epidermis and neuromuscular attachment

We have found that complete or strong loss of *lin-14* function results in severe defects in ventral nerve and muscle organization, reminiscent of mutants lacking components of the ECM or CeHD epidermal cell-matrix adhesions. Extensive studies in *C. elegans* have delineated pathways required for the initial formation of cell matrix adhesions in embryonic epidermis.^45^ Complete loss of function in components of cell matrix adhesions such as Plectin/*vab-10A*^46^ or Intermediate Filaments/*ifa-2*^47^ results in severe muscle detachment, epidermal morphology defects, neuronal mispositioning and embryonic lethality. Similar phenotypes are seen in mutants lacking matrix proteins such as F-spondin/*spon-1*^48^ or Peroxidasins/*pxn-2*.^49^ The muscle detachment and VNC mispositioning defects in *lin-14* mutants arise in the mid L1 stage and are restricted to the ventral midbody. Thus, LIN-14 is not essential for CeHD biogenesis but plays a specific role in assembly of CeHDs during L1-specific maturation of ventral epidermis.

The defects in nerve and muscle attachment in *lin-14* mutants display several notable features. First, muscle detachment appears to precede VNC disorganization, in that the latter is not seen without the former. In other muscle attachment (*mua*) mutants, muscle detachment defects are generally irreversible, although *mua* phenotypes can be suppressed by loss of muscle contractility, indicating they are caused by fragile muscle-epidermal or intra-epidermal attachment.^50^ It would be interesting to examine whether the muscle detachment in *lin-14* mutants is also contractility-dependent. Second, in *lin-14* mutants, VNC mispositioning appears to preferentially affect embryonic neurons in that the severity of defects does not increase after post-embryonic neuron formation. Moreover, mild mispositioning of VNC motor neuron somas in the L1 stage appears to be corrected over subsequent larval stages. As yet, we do not know whether or how the postembryonic neurons integrate with the mispositioned embryonic neurons.

### LIN-14 and ventral epidermis reorganization

The postembryonic maturation of the ventral body epidermis in the L1 stage has been described in detail by Podbilewicz et al.^12^ in fixed samples and by Bone et al.^13^ in live animals. In normal development, disassembly of ventral CeHDs in the ventrally retracting juvenile ventral epidermis (P cells), is rapidly followed by assembly of new CeHDs in the ventrally intercalating mature ventral epidermis (V-derived hyp7). The seamless transition from juvenile to mature CeHDs on the ventral epidermis suggests the newly generated CeHD arrays could be templated on the epidermal-muscle ECM or muscle itself. As yet, the molecular mechanism of ventral epidermal maturation is not well understood. Our studies indicate that the CeHD disassembly in P cells is independent of CeHD reassembly in hyp7, as *lin-14* mutants are defective in the latter but not the former. Thus, LIN-14 does not appear to be required for the coordination of CeHD assembly during embryogenesis or disassembly in the retracting P cells from the epidermis, but is required for the maturation of ventral epidermis. The generally normal dorsal nerve and muscle organization in *lin-14* mutants may reflect the dorsoventral asymmetry of epidermal development, in that dorsal (hyp7) epidermis does not undergo overt remodeling in the L1 stage. We speculate that *lin-14,* as a key temporal regulator, maintains cellular competence that enables new CeHD formation in regions previously occupied by P cells. It will be important to identify downstream effectors of LIN-14 in the maturation of ventral epidermis.

### LIN-14 regulation of CeHD assembly in maturing ventral epidermis

How might LIN-14 regulate local CeHD assembly? LIN-14 might act directly on CeHD gene expression in the L1, possibly in Vn.a cells prior to their fusion with hyp7. LIN-14 may also act separately in the ventral vs. dorsal epidermis, as increased CeHD levels were observed in *lin-14* mutant dorsal epidermis. LIN-14 may be permissive for expression of CeHD assembly genes in the L1 in response to other spatial cues. LIN-14 regulates many target genes such as *ins-33*^51^ and *nlp-45*.^31^ Genome-wide chromatin immunoprecipitation and sequencing (ChIPseq) have revealed many additional candidate LIN-14 targets^31^ including *vab-10*. Promoters of such genes show higher binding with LIN-14 in L1 compared with L2, suggesting that these genes may be temporally regulated by LIN-14. BEN domain proteins frequently act as transcriptional repressors, so LIN-14 could also repress L2 specific programs that inhibit post-embryonic CeHD assembly.

The failure of ventral CeHD assembly in *lin-14* mutants could also be an indirect result of the transformation of L1-like seam divisions to L2-like divisions. As a consequence of their fate transformations, *lin-14* mutant L1 seam cells generate ∼12 V grand-daughters (Vn.xa cells) instead of the six Vn.a daughters that normally fuse with dorsal epidermal hyp7 syncytium. In wild type, V descendants fuse with hyp7 in L1 and L2 stages, but their fusion in *lin-14* mutants may be delayed relative to normal L1 Vn.a daughters due to the extra cells or steric constraints. The delay in fusion of Vn.xa cells with hyp7 in *lin-14* L1s means that dorsal and ventral hyp7 regions may be less interconnected.

Previous studies of the assembly of new CeHDs during later postembryonic growth^11^ have suggested that CeHDs grow by intercalation of new CeHDs into existing arrays, a phenomenon that is coordinated throughout the epidermis. Although it is unclear if this model applies to the assembly of new CeHDs during ventral reorganization in the L1, it suggests CeHDs may not be trafficked as units but rather assemble in response to a combination of local and global cues. Possibly, the local cue involves interaction with muscles via the basal receptor LET-805.

In conclusion, we have uncovered the BEN domain protein LIN-14’s role in tissue interactions during development. Given the recent findings that LIN-14 is a member of a conserved protein family, our observations raise the question of whether other BEN domain proteins have analogous roles. In mammals, initial embryonic HD formation has been extensively characterized,^52^^,^^53^^,^^54^^,^^55^ as has mechanisms of HD turnover during wound healing.^56^ An important future direction will be to assess whether BEN domain proteins may regulate mammalian HD dynamics in skin homeostasis or wound healing.

### Limitations of the study

We have shown that LIN-14’s function in ventral neuromuscular positioning requires the DNA binding BEN domain. However, we do not know whether LIN-14 directly or indirectly regulates expression of CeHDs, or other components required for CeHD assembly. This limitation is partly due to the complex gene structure of *vab-10* and *let-805.* We observed defective assembly of CeHD during L1 ventral tissue reorganization in *lin-14* mutants. Yet, we do not know the cellular source of CeHD, which is partly due to technical difficulty in expressing and manipulating large proteins, such as VAB-10 and LET-805. Our study also did not address the epidermal cell type specificity of LIN-14 requirement for CeHD formation in the maturing ventral epidermis.

## Resource availability

### Lead contact

Further information and requests for resources and reagents should be directed to and will be fulfilled by Yishi Jin (yijin@ucsd.edu).

### Materials availability

All reagents generated in this study are available from the lead contact.

### Data and code availability

• All original, unprocessed microscopy images and raw quantitative data are available in Figshare: http://doi.org/10.6084/m9.figshare.27984296.v1.
• This paper does not report original code.
• Any additional information required to reanalyze the data reported in this paper is available from the lead contact upon request.

## Acknowledgments

We thank Kota Mizumoto for advice on genome editing and AID plasmids, David Reiner and Jordan Ward for advice on TIR1 transgenes, Oliver Hobert for pAB1 plasmid, HaoSheng Sun for strain HUG60, Karina Cerda and Kavanaugh Kaji for technical assistance, and members of the Jin and Chisholm labs for helpful discussion. We acknowledge Wormbase for valuable resource. Some strains were provided by the CGC, which is funded by NIH Office of Research Infrastructure Programs (P40, OD010440). E.J.J. was a trainee in the Neural Circuits Postdoctoral Training Program at UCSD (T32NS007220) and a recipient of an AP Giannini Foundation Postdoctoral Fellowship. This work was supported by grants from 10.13039/100000002NIH (R35 GM134970 to A.D.C. and R35 NS127314 to Y.J.).

## Author contributions

E.J.J., A.D.C., and Y.J. conceived the project. E.J.J. performed all experiments. Y.B.Q. generated strains and plasmids and made initial observations. E.J.J., Y.J., and A.D.C. secured funding, analyzed data, and wrote the paper. All authors reviewed and edited the paper.

## Declaration of interests

The authors declare no competing interests.

## STAR★Methods

### Key resources table

**Table:** 

REAGENT or RESOURCE	SOURCE	IDENTIFIER
**Bacterial and virus strains**		

*Escherichia coli*	*Caenorhabditis* Genetics Center	OP50; RRID: WB-STRAIN:WBStrain00041969

**Chemicals, peptides, and recombinant proteins**		

Indole-3-acetic acid, 98+%	Thermo Fisher	A10556.14
Levamisole	Sigma	L9756
Agar	BD	214040

**Critical commercial assays**		

QIAprep Spin Miniprep Kit	Qiagen	27104
Zymoclean Gel DNA Recovery Kit	Zymo Research	D4001/D4002
QIAGEN Plasmid Plus Midi Kit	Qiagen	12943
TURBO™ DNase (2 U/μL)	Thermo Fisher	AM2238
TOPO-TA cloning kit	Thermo Fisher	K250020
Gateway LR Clonase II Enzyme mix	Invitrogen	12538–120
Gibson Assembly Master Mix	NEB	E2611
DreamTaq Green DNA Polymerase	Thermo Fisher	EP0711
Phusion High-Fidelity DNA Polymerase	Thermo Fisher	F530L
ExoSAP-IT PCR Product Cleanup Reagent	Thermo Fisher	78201.1.ML
Superscript III Reverse Transcriptase	Thermo Fisher	18080044
TRIzol reagent	Thermo Fisher	15596026

**Deposited data**		

Raw Image Data and Spreadsheets	Figshare	Figshare: https://doi.org/10.6084/m9.figshare.27984296.v1

**Experimental models: Organisms/strains**		

*C. elegans*: N2 (Bristol) Wild-type	*Caenorhabditis* Genetics Center	RRID: WB-STRAIN:WBStrain00000001
*C. elegans*: *Pacr-2-GFP(juIs14) IV*	Hallam et al.^57^	CZ631
*C. elegans*: *Pacr-2-GFP(juIs14) IV; lin-14(ma135) X/szT1 I,X*	This study	CZ7447
*C. elegans*: *juIs14 [Pacr-2-GFP] IV; lin-14(cc2841ju1945) X*	This study	CZ30641
*C. elegans*: *Pacr-2-GFP(juIs14) IV; lin-14(n179ts) X*	This study	CZ7451
*C. elegans*: *juIs14 [Pacr-2-GFP] IV; lin-14(cc2841ju1945) X/szT1 I,X; juEx8440[lin-14 fosmid WRM0632cE08 + Pttx-3-mScarlet-I]*	This study	CZ30862
*C. elegans*: *juIs14 [Pacr-2-GFP] IV; lin-14(cc2841ju1945) X/szT1 I,X; juEx8443[lin-14 fosmid WRM069dF11 + Pttx-3-mScarlet-I]*	This study	CZ30863
*C. elegans*: *Punc-25-GFP juIs76 II*	Huang et al.^58^	CZ13799
*C. elegans*: *juIs76 [Punc-25-gfp] II; lin-14(ma135) X/szT1*	This study	CZ7445
*C. elegans*: *juIs76 [Punc-25-GFP] II; lin-14(cc2841ju1945) X/szT1 I,X*	This study	CZ30013
*C. elegans*: *lin-14(cc2841ju1945) X; juIs236 [Punc-25-wcherry::RAB-3 + Pttx-3-DsRed] II*	This study	CZ30014
*C. elegans*: *lin-14::GFP(cc2841) X; juIs236[Punc-25-wcherry::RAB-3 + Pttx-3-DsRed] II*	This study	CZ27888
*C. elegans*: *unc-54::GFP(cc2856) I; unc-17::mKate2(ot907))IV; lin-14(cc2841ju1945)/szT1 X*	This study	CZ30563
*C. elegans*: *unc-54::GFP(cc2856) I; unc-17::mKate2(ot907) IV; lin-14(cc2841ju1945) X*	This study	CZ30739
*C. elegans*: *lin-14(ju1965 [lin-14::AID∗::mScarlet-I]) X*	This study	CZ29654
*C. elegans*: *Pacr-2-GFP(juIs14) IV; lin-14(ju1965 [lin-14::AID∗::mScarlet-I]) X*	This study	CZ29700
*C. elegans*: *reSi7[rgef-1p-TIR1::F2A::mTagBFP2::AID∗::NLS::tbb-2 3′UTR] I; juIs14 [Pacr-2-GFP] IV; lin-14(ju1965 [lin-14::AID∗::mScarlet-I]) X*	This study	CZ29789
*C. elegans*: *reSi3[unc-54p-TIR1::F2A::mTagBFP2::AID∗::NLS::tbb-2 3′UTR] I; juIs14 [Pacr-2-GFP] IV; lin-14(ju1965 [lin-14::AID∗::mScarlet-I]) X*	This study	CZ29788
*C. elegans*: *reSi2 [col-10p-TIR1::F2A::mTagBFP2::AID∗::NLS::tbb-2 3′UTR] II; juIs14 [Pacr-2-GFP] IV; lin-14(ju1965 [lin-14::AID∗::mScarlet-I]) X*	This study	CZ29970
*C. elegans*: *reSi12 [ges-1p-TIR1::F2A::mTagBFP2::AID∗::NLS::tbb-2 3′UTR] II; juIs14 [Pacr-2-GFP] IV; lin-14(ju1965 [lin-14::AID∗::mScarlet-I]) X*	This study	CZ29945
*C. elegans*: *wrdSi47 [dpy-7p-TIR1::F2A::mTagBFP2::AID∗::NLS::tbb-2 3′UTR] (I:-5.32); juIs14 [Pacr-2-GFP] IV; lin-14(ju1965 [lin-14::AID∗::mScarlet-I]) X*	This study	CZ30195
*C. elegans*: *unc-54::GFP(cc2856) I; reSi2 [col-10p-TIR1::F2A::mTagBFP2::AID∗::NLS::tbb-2 3′UTR] II; lin-14(ju1965 [lin-14::AID∗::mScarlet-I]) X*	This study	CZ30490
*C. elegans*: *juIs14 [Pacr-2-GFP] IV; lin-14(cc2841ju1945) X;/szT1 I,X; juEx8435[Pcol-10-lin-14(gDNA)::gfp+Pinx-6-TagRFP]*	This study	CZ30871
*C. elegans*: *juIs1[Punc-25-SNB-1::GFP S65T I167 +lin-15(+)] IV; lin-14(ju1965 [lin-14::AID∗::mScarlet-I]) X*	This study	CZ29698
*C. elegans*: *reSi7[rgef-1p-TIR1::F2A::mTagBFP2::AID∗::NLS::tbb-2 3′UTR] I; juIs1[Punc-25-SNB-1::GFP S65T I167 +lin-15(+)] IV; lin-14(ju1965 [lin-14::AID∗::mScarlet-I]) X*	This study	CZ29760
*C. elegans*: *reSi3[unc-54p-TIR1::F2A::mTagBFP2::AID∗::NLS::tbb-2 3′UTR] I; juIs1[Punc-25-SNB-1::GFP S65T I167 +lin-15(+)] IV; lin-14(ju1965 [lin-14::AID∗::mScarlet-I]) X*	This study	CZ29758
*C. elegans*: *reSi2[col-10p-TIR1::F2A::mTagBFP2::AID∗::NLS::tbb-2 3′UTR] II; juIs1[Punc-25-SNB-1::GFP S65T I167 +lin-15(+)] IV; lin-14(ju1965 [lin-14::AID∗::mScarlet-I]) X*	This study	CZ29822
*C. elegans*: *reSi12[ges-1p-TIR1::F2A::mTagBFP2::AID∗::NLS::tbb-2 3′UTR] II; juIs1[Punc-25-SNB-1::GFP S65T I167 +lin-15(+)] IV; lin-14(ju1965 [lin-14::AID∗::mScarlet-I]) X*	This study	CZ29871
*C. elegans*: *lin-14(n179ts) X; scm::gfp(wIs54) V*	This study	CZ11076
*C. elegans*: *vab-10a::GFP(cas602) I*	Yang et al.^44^	GOU2043
*C. elegans*: *vab-10a::GFP(cas602) I; lin-14(cc2841ju1945) X*	This study	CZ30746
*C. elegans*: *let-805::GFP(ju1448) III*	Gotenstein et al.^43^	CZ23945
*C. elegans*: *let-805::GFP(ju1448) III; lin-14(cc2841ju1945) X*	This study	CZ30463
*C. elegans*: *vab-10a::GFP(cas602) I; reSi2 [col-10p-TIR1::F2A::mTagBFP2::AID∗::NLS::tbb-2 3′UTR] II; lin-14(ju1965 [lin-14::AID∗::mScarlet-I]) X*	This study	CZ30411
*C. elegans*: *reSi2 [col-10p-TIR1::F2A::mTagBFP2::AID∗::NLS::tbb-2 3′UTR] II; let-805::GFP(ju1448) III; lin-14(ju1965 [lin-14::AID∗::mScarlet-I]) X*	This study	CZ30609
*C. elegans*: *vab-10a::GFP(cas602) I; lin-14(cc2841ju1945 [lin-14::AID∗-exon5-stop::gfp]) X;/szT1 I,X; juEx8435[Pcol-10-lin-14(gDNA)::gfp+Pinx-6-TagRFP]*	This study	CZ30947
*C. elegans*: *vab-10a::GFP(cas602) I; ncIs13[AJM-1::GFP] II; lin-14(cc2841ju1945) X/szT1 I,X*	This study	CZ30618
*C. elegans*: *Punc-25-GFP(juIs76) II; lin-14(ma135) X/szT1 I,X; Prgef-1-lin-14(juEx2377)*	This study	CZ10845
*C. elegans*: *Pacr-2-GFP(juIs14) IV; lin-14(syb5772) X;/szT1 I,X*	This study	CZ31094
*C. elegans*: *Punc-25-GFP(juIs76) II; lin-14(cc2841ju1945) X*	This study	CZ31120
*C. elegans*: *Pflp-13-GFP(juIs145) II; lin-14(cc2841ju1945) X*	This study	CZ31121

**Oligonucleotides**		

Primer to amplify AID∗ repair template from AID∗-mTagBFP2 plasmid, to generate *lin-14(cc2841ju1945)*: tagccaaaaaacatttaaacatttaattaatgagaatcatgctttttttcag AGGAGCCGGAGCCCCTAAAGATCCAGCCAAACCTCCGG	This study	SD20097
Primer to amplify AID∗ repair template from AID∗-mTagBFP2 plasmid, to generate *lin-14(cc2841ju1945)*: CATAGGCAGTATCAAATTCACTGCTTGTCGAAGATCGGTTA CTTCCTTCACGAACGCCGCCGCCTCCGGGCCACCGCTTG	This study	SD20098
Genotyping primer for *lin-14(cc2841ju1945)*: GATCTGCCTGGAACGTCTTCG	This study	SD20051
Genotyping primer for *lin-14(cc2841ju1945)*: GCTGTAGAGTTGGCTGTGCTG	This study	SD20054
Primer for RT-PCR analysis, to amplify exons 4 to 6: GATCTGCCTGGAACGTCTTCG	This study	SD20051
Primer for RT-PCR analysis, to amplify exons 4 to 6: GTAATGTCGGCGATGCTGGTT	This study	SD20169
Primer for RT-PCR analysis, to amplify exon 7 to GFP in *lin-14(cc2841ju1945)*: GACGTAATTGGCGATGGCAG	This study	SD20020
Primer for RT-PCR analysis, to amplify exon 7 to GFP in *lin-14(cc2841ju1945)*: CACCTTCAAACTTGACTTCAGC	This study	SD21417
Primer to amplify AID∗::mScarlet-I repair template from pCZGY3644, to generate *lin-14(ju1965)*: CTTATCCGAACTAAAGTGGAATCACAATCTCCTCCTCTT ccggtagaaaaaATGGGAGCCGGAGCCCCTAAAGATCCAGCC	This study	SD20223
Primer to amplify AID∗::mScarlet-I repair template from pCZGY3644, to generate *lin-14(ju1965)*: gaaggatgactcgaaaaattggcattCTATTGTGGACCTTGagggca CTTGTAGAGCTCGTCCATTCCTCCGGTGGAGTGA	This study	SD20224
Genotyping primer for *ju1965*: TGAACGGAGCTGGTTTGATAAGG	This study	SD21397
Genotyping primer for *ju1965*: GAGTGTACGGAAGAGTCAATCCAG	This study	SD21398
Primer to amplify *Pcol-10* to clone pCZGY3639: tatagggcgaattgggtaccGATCTTCATCCCTTCAAC	This study	SD20492
Primer to amplify *Pcol-10* to clone pCZGY3639: ggtcctttggccaatcccggAAGCCAGGTACCTTATTC	This study	SD20493
Primer to amplify gtwy-backbone to clone pCZGY3639: tacctggcttCCGGGATTGGCCAAAGGAC	This study	SD20500
Primer to amplify gtwy-backbone to clone pCZGY3639: gatgaagatcGGTACCCAATTCGCCCTATAG	This study	SD20501
Primer to amplify gtwy-mScarlet-I to clone pCZGY3644: gttcgtgaagGTCTCCAAGGGAGAGGCC	This study	SD20198
Primer to amplify gtwy-mScarlet-I to clone pCZGY3644: ctccggctccCATGATATCAATACCATGGTACATCACC	This study	SD20199
Primer to amplify AID∗ to clone pCZGY3644: tgatatcatgGGAGCCGGAGCCCCTAAAG	This study	SD20200
Primer to amplify AID∗ to clone pCZGY3644: ccttggagacCTTCACGAACGCCGCCGC	This study	SD20201
crRNA for *lin-14(cc2841ju19450)*: 5′-AGAAAATTAGAAACATTCAT-3′**GGG** (for all crRNAs: **PAM site is in bold** and not included in the crRNA sequence)	This study	SD21405
crRNA for *lin-14(ju1965)*: 5′- ATTGTGGACCTTGAAGAGG-3′**AGG**	This study	SD21406

**Recombinant DNA**		

*AID-mTagBFP2-loxP_myo2_neoR-loxP*	Hendi et al.^59^	RRID: Addgene_194055
pCZGY416: *pCR8-lin-14::GFP*	This paper	
pCZGY2262: *Pflp-13-GTW*	Noma et al.^60^	
pCZGY3600: *gtwy-mScarlet-1::unc-54 3′UTR*	This paper	
pCZGY3639: *gtwy-Pcol-10*	This paper	
pCZGY3640: *Pcol-10-lin-14::GFP*	This paper	
pCZGY3644: *gtwy-AID∗::mScarlet-I::unc-54 3′UTR*	This paper	
pCZGY3601: *Pttx-3-mScarlet-1::unc-54 3′UTR*	This paper	
*inx-6p(2TAAT-deletion)-tagRFP::unc-543′ UTR*	Bhattacharya and Hobert^61^	
pRF4 (*rol-6(su1006dn)*))	Mello et al.^62^	
Fosmid WRM0632cE08	*C. elegans* Vancouver fosmid library	
Fosmid WRM069dF11	*C. elegans* Vancouver fosmid library	
pCZGY936: *Prgef-1-lin-14*	This paper	

**Software and algorithms**		

Prism 10	GraphPad	https://www.graphpad.com
FIJI ImageJ	Schindelin et al.^63^	https://imagej.net/software/fiji/
ImarisViewer 10.1.1	Oxford Instruments	https://imaris.oxinst.com/imaris-viewer
A plasmid Editor (ApE)	Davis and Jorgensen^64^	https://jorgensen.biology.utah.edu/wayned/ape/

### Experimental model and study participant details

#### *C. elegans* genetics

All worm strains and crosses were maintained at room temperature (∼23°C) on nematode growth media with *Escherichia coli* OP50, unless specified otherwise. *lin-14* mutant strains were constructed using *szT1* balanced males and *lin-14* mutant hermaphrodites. *lin-14* mutants were genotyped visually for morphological phenotypes, and *lin-14(cc2841ju1945)* was verified by PCR genotyping.

### Method details

#### Molecular cloning

pCZGY3639 [gtwy-*Pcol-10*] was made by Gibson assembly of two PCR products: *Pcol-10* (1137 bp upstream of ATG) amplified from N2 lysate using primers SD20492 and SD20493, and destination vector backbone amplified from pCZGY2262 [gtwy-*Pflp-13*] using primers SD20500 and SD20501. pCZGY416 was made by subcloning an *Eco*RI fragment (6,612 bp) containing *lin-14::GFP* from pB14R-GFP^57^ into pCR8, and Gateway LR reaction was then performed between pCZGY416 [*pCR8-lin-14::GFP*] and pCZGY3639 to create pCZGY3640 [*Pcol-10-lin-14::GFP*]. pCZGY3644 [gtwy-AID^∗^::mScarlet-I] was made by Gibson assembly of AID^∗^, amplified from AID^∗^-tagBFP2 plasmid (RRID: Addgene_194055) using primers SD20200 and SD20201, and gtwy-mScarlet-I, amplified from pCZGY3600 [gtwy-mScarlet-I] using primers SD20198 and SD20199.

#### Transgene construction and germ line transformation

Fosmid DNAs were prepared from the *C. elegans* Vancouver fosmid library as described.^58^ *lin-14* containing fosmids WRM0632cE08, and WRM069dF11 were injected at 1 ng/μL into N2. pCZGY3601 [*Pttx-3*-mScarlet-I] was used as coinjection marker at 100 ng/μL pCZGY3640 [*Pcol-10*-*lin-14*::*GFP*] was injected at 5 ng/μL into N2, and pAB1 [*Pinx-6*-tagRFP] was used as coinjection marker at 100 ng/μL. Multiple independent lines were obtained. Two fosmid lines (*juEx8440[WRM0632cE08] and juEx8443 [WRM069dF11]*) and one transgene expressing *lin-14::gfp* in epidermis (*juEx8435[Pcol-10-lin-14::GFP])* were crossed into CZ30030 (*juIs14[Pacr-2-GFP]; lin-14(cc2841ju1945)/szT1*) to generate CZ30862 (*juIs14; lin-14(cc2841ju1945)/szT1; juEx8440)*, CZ30863 (*juIs14; lin-14(cc2841ju1945)/szT1; juEx8443)* and CZ30871 (*juIs14; lin-14(cc2841ju1945)/szT1; juEx8435)*, respectively. We scored cross progeny males for rescue of *lin-14(cc2841ju1945)*. *lin-14(cc2841ju1945)* males are uncoordinated, small, and mating defective; *lin-14* fosmid and epidermal *Pcol-10-lin-14::gfp* transgenes rescued morphological defects of *lin-14(cc2841ju1945)* males and the transgene-containing males were able to mate. We further scored VNC mispositioning in transgenic males by scoring the number of cholinergic motor neuron somas positioned >1 soma distance from the ventral midline.

#### CRISPR/Cas9 mediated genome editing

We used the melting method^59^ to generate *lin-14(cc2841ju1945)* and *lin-14(ju1965)* and used CZ27888 *juIs236[Punc-25::wCherry::RAB-3]; lin-14::GFP(cc2841) X* as host for editing.

*lin-14(cc2841ju1945)* had a 197 bp insertion containing AID∗ after the first nucleotide of exon 5 of *lin-14(cc2841).* AID^∗^ was amplified from AID^∗^-mTagBFP2 plasmid (RRID: Addgene_194055) using primers SD20097 and SD20098, then gel purified using ZYMO gel extraction kit. crRNA SD21405 was used and RNP complex was prepared as described in.^59^ Injection mix was prepared with 0.5 μM RNP complex, 50 ng/μL repair template, 25 ng/μL pRF4, and RNase free water. The injection mix was centrifuged at 25,200 g for 10 min, then injected into CZ27888. F_1_ Roller animals were selected and genotyped by PCR amplification (primers SD20051 and SD20054; WT product is 384 bp, and *lin-14(cc2841ju1945)* product is 574 bp). The desired edits were verified in the final candidates by Sanger sequencing with primer SD20054.

*lin-14(ju1965)* contained AID^∗^::mScarlet-I insertion 12 nucleotides before the stop codon, the same location as GFP in *lin-14(cc2841)*. The two linkers on AID^∗^::mScarlet-I are: ccggtagaaaa on 5′, and tgccct on 3’. The AID^∗^::mScarlet-I repair template was amplified from plasmid pCZGY3644 (gtwy-AID^∗^::mScarlet-I) with primers SD20223 and SD20224, then gel purified. crRNA SD21406 was used, and the RNP complex and injection mix were prepared as described above. The injection mix was injected into N2. F_1_ roller animals were selected and genotyped by PCR amplification (primers SD21397 and SD21398; WT product is 502 bp, and *lin-14(ju1965)* product is 1513 bp). The desired edits were verified by Sanger sequencing.

#### RT-PCR

RT-PCR was performed on strains N2, CZ30261 and CZ30563. 3–4 plates of worms were used per genotype for RNA extraction. In brief, total worm RNA was extracted using TRIzol, followed by DNase treatment using TURBO DNase, then RNA was repurified using phenol chloroform. 1 μg of RNA was reverse transcribed to synthesize cDNA using Superscript III RT. Exons 4 to 6 of *lin-14* were amplified by PCR using primers SD20051 and SD20169, and Sanger sequencing of the resulting cDNAs showed 197 nt insertion after the first nucleotide of exon 5. Exon 7 to GFP was amplified using primers SD20020 and SD21417; Sanger sequencing of the resulting cDNAs showed no changes.

#### Determining larval stages

L1 stages were determined using DIC microscopy based on P cell nuclei migration and 4 cell divisions as described in Sulston and Horvitz (1977). We defined ‘early L1’ as prior to P nuclear migration (0–7.5 h post hatching), ‘mid L1’ from anterior P nuclei migration (7.5–12 h) to 2^nd^ (P.xx) divisions, and ‘late L1’ from 3^rd^ to 4^th^ (P.xxx to P.xxxx) divisions (12–16 h). *lin-14(ma135)* or *lin-14(cc2841ju1945)* mutants do not generate L1 alae and therefore late L1 was not distinguished from early L2; mutant animals were grouped together as “late L1-L2”. *lin-14* mutants with ventral protrusion(s) and absence of fertilized eggs were categorized as L4.

To determine Q and V cell divisions in early L1, we used the seam cell reporter *wIs54[scm::gfp]*,^60^ which is also expressed in Q. The number and positions of Q and V cells were manually quantified using fluorescence compound microscope AxioImager M2.

#### Auxin mediated LIN-14 depletion

5 mM IAA solution was freshly prepared before use from 400 mM IAA stock (IAA dissolved in ethanol; kept at −20°C in dark for up to 1 month) and M9. For auxin treatment throughout development, IAA plates were prepared as follows: 300 μL of 5 mM IAA solution was pipetted evenly on a seeded plate, then kept in dark at room temperature to let the solution dry for 0.5–1 h. 5 L4s or YAs were placed on each plate on day 1, then were removed from the plate on day 2. Efficient LIN-14::mScarlet-I depletion was validated in early L1 progeny (∼20 animals from each auxin plate) on day 2, and then L4s were scored on day 3–4. The scored progeny are not the same animals tested for LIN-14::mScarlet-I depletion. All plates were kept in the dark at room temperature (∼23°C) at all times.

For short-term auxin treatment during embryo or L1 development, 20 gravid adults were placed on seeded plate for 1 h to lay embryos, then were removed. Then 300 μL of 5 mM IAA solution was pipetted directly onto each plate for auxin treatment, and were kept in dark at RT. To terminate auxin treatment, embryos or larvae were transferred to seeded plate without auxin, and were kept in light on the bench. Progeny were scored 24 h after egg-laying (late L1 - early L2) for VNC mispositioning. At each time point, plates were examined for hatched progeny and absence of embryos to determine the hatching period of 6 h–9.5 h after egg laying: 1–5 hatched progeny were observed at 6 h after egg laying, and no unhatched embryos were observed at 9.5 h after egg laying.

Similar methods were used to assess LIN-14::AID∗::mScarlet-I depletion kinetics, with the following modifications: 5 mM IAA solution was added directly to each plate when ∼10 hatched progeny were observed. No auxin control was included per genotype. All plates were kept in dark at RT, and at each time point indicated (30, 60, 105, 135, 180 min), ∼10–15 animals per condition were mounted on agar pad on microscope slide, and both DIC and mScarlet-I images focusing on the respective tissue nuclei were taken using AxioImager M2. Animals for two time points were used per auxin plate, to reduce light exposure.

#### Temperature shift experiments

*lin-14(n179ts)* mutants were maintained for over 2 generations in either 15°C (permissive temperature) or 25°C (restrictive), then scored for VNC mispositioning using *juIs14*. For short interval temperature shifts, the temperature-sensitive mutant *lin-14(n179ts)* raised at 15°C over 2 generations was used. 20 adult mutants were placed on each plate for 2 h to lay eggs at 15°C, then were removed. Then these plates were shifted from 15°C to 25°C, then back to 15°C during the time periods indicated in Figure 2H. For the 25°C control condition, the plates were transferred from 15°C to 25°C after removing the 20 adults, for the remainder of the experiment. Progeny were scored at 36–38 h after egg laying (early L2) for VNC mispositioning.

#### Locomotion assays

For locomotion on agar plate, 10 animals were placed in the middle of NGM plate with OP50. 1 min or 1 h after the worms were placed on the plate, images were taken with an iPhone through an eyepiece of Leica MS5 dissecting microscope at 0.63X magnification. Total distance traveled in 1 min was quantified by tracing an animal’s travel path using ImageJ Fiji.

For thrashing assay in liquid, individual L4 animals were placed in a drop of M9 on NGM plate, then the number of body bends was counted per minute. Representative images were taken from a video recording of thrashing behavior using an iPhone through an eyepiece of Leica MS5 at 2X magnification.

#### Microscopy and image processing

Animals were mounted on 4% agar pads with 2.5 mM levamisole. Worms were observed using Zeiss AxioImager M2 compound microscope with Zeiss HXP 120C, with 20X, 40X, or 63X Plan-Apochromat objectives. Filters used: Zeiss filter set 38 HE for GFP, Chroma 49008 for mCherry, mScarlet-I and RFP, and Zeiss filter set 47 HE for CFP or BFP. Representative images were taken using Zeiss Axiocam 705 monochrome camera, ZEN3.1 pro and the following exposure times: 10 ms for DIC and 200 ms for fluorescence. Fiji was used to open the czi files, convert to RGB then to save as tif. All worms in figures are oriented with left lateral side up, with anterior to the left and dorsal to the top, unless specified otherwise in the figure legends. When comparing fluorescence intensity between genotypes, fluorescence was equally adjusted between all genotypes.

Confocal images were acquired using Zeiss LSM800 confocal microscope with a 63X Plan-Apochromat objective. For AJM-1::GFP and VAB-10A::GFP imaging, 488 nm laser power was set at 2.6% and detector gain at 650V, and 4 line averages were performed during image acquisition. For all confocal images, z stacks spanning the entire worm were acquired with an increment of 0.3 μm. ImarisViewer was used to visualize 3D projections of acquired z stack images.

### Quantification and statistical analysis

#### Quantification of ventral nerve cord (VNC) and muscle detachment

L4-adult, L1 (early, mid or late L1) or early L2 animals (precise stage is indicated in figures and figure legends) expressing ventral cord motor neuron markers (*juIs14, juIs76, juIs145, ot907*) were mounted on 4% agar pads with 2.5 mM levamisole, and scored visually under Zeiss AxioImager M2 with 40X Apochromat objective. DIC was used to determine L1 stage of each animal; GFP filter was used to score VNC (*juIs14)* or muscle detachment (*cc2856),* and RFP filter for *ot907*. VNC detachment was scored in two ways: “# mispositioned somas” was determined by visually counting per animal the number of cholinergic motor neuron somas (using *juIs14* as reporter) that are positioned >1 soma length away (lateral and dorsally misplaced) from the ventral midline; “% of VNC detachment” was quantified by ‘# of animals displaying 2 or more mispositioned somas’/‘total number of animals scored’. “# misguided and ectopic axons” was quantified by visually counting the number of ventral and commissure axons displaying >1 extra branch.

“Muscle detachment” was visually scored as animals displaying ventral muscles (UNC-54::GFP(*cc2856)* reporter) with >1 cholinergic soma length (*juIs14)* away from ventral midline. “% muscle-only detachment” was scored by ‘# of animals displaying muscle detachment’/‘total number of animals scored’. “% muscle and VNC detachment” was scored by ‘# of animals displaying muscle and VNC (UNC-17::mKate(*ot907)* reporter) detachment together’/‘total number of animals scored’.

#### Quantification of LIN-14::AID∗::mScarlet-I depletion

LIN-14::AID∗::mScarlet-I levels were quantified from compound microscope images of early L1 animals focusing on the nuclei of motor neurons, dorsal body wall muscles, intestine, or lateral epidermis. Mean fluorescence intensity of ROI (a circle with ∼1.5 μm diameter) inside and right outside each nucleus were measured, and the outside levels (background levels) were subtracted from inside nucleus levels for every nucleus measured. This background subtracted mean fluorescence intensity of auxin treated conditions were normalized to no-auxin controls. Tissue specific nuclei were determined using DIC to not be biased by LIN-14::AID∗::mScarlet-I levels. Neurons: motor neurons DD4, DB6 and DA6 were quantified per individual. Dorsal body wall muscles and intestine: 3 nuclei were quantified per individual (1 anterior, 1 mid body, 1 posterior). Lateral epidermis: 4 nuclei were quantified per individual (1 anterior, 2 mid body, 1 posterior).

#### Quantification of body length and ventral protrusions

Brightfield images of entire animal body of L4s were taken using Zeiss AxioImager M2 with 20X objective. For body length measurement, Fiji was used to draw a line from mouth tip to tail, in the middle of the animal body, following the body curvature. For ventral protrusion quantification, the number of protrusions along ventral midline were manually counted.

#### Quantification of hemidesmosome expression

To quantify the extent and fluorescence intensity levels of VAB-10A::GFP and LET-805::GFP, images were taken using compound fluorescence microscope Zeiss AxioImager M2 and 40X objective, with 50 ms exposure time for LET-805 and 200 ms exposure time for VAB-10A::GFP, and 10 ms for DIC. L1 stages were determined using DIC. Control and *lin-14* mutants were imaged under identical conditions. “% of ventral body expression of LET-805::GFP or VAB-10A::GFP” = “sum of the total lengths of expression along ventral epidermis, from nose tip to anus”/“total length of animal, from nose tip to anus”. Lengths were measured by manually drawing lines along the ventral epidermis using Fiji.

Expression levels of hemidesmosome markers were measured using FIJI ImageJ, by drawing a line (∼20 μm) on dorsal epidermis (opposite of gonad) or anterior epidermis (ventral, adjacent to metacorpus), then measuring the mean fluorescence intensity (AU). Fluorescence intensities were normalized to control.

#### Statistical analyses and reproducibility

Unpaired t-test was used to compare two conditions. Multiple conditions were compared using One way ANOVA followed by a multiple comparison test as stated in each Figure legend (ns *p* > 0.05, ∗*p* < 0.05, ∗∗*p* < 0.01, ∗∗∗*p* < 0.001, ∗∗∗∗*p* < 0.0001). Chi-squared test followed by Marascuilo procedure were used to compare % of categorical phenotypes between conditions (ns *p* > 0.05, #*p* < 0.05). n is total number of animals tested per condition, each datapoint represented as an open circle is an individual animal, mean ± SEM is indicated as a bar or line, and datapoints from multiple experimental repeats were pooled together. The exact value of n and statistical analysis performed can be found in the Figure legends.
