# Supplementary material for: The BEN domain protein LIN-14 coordinates neuromuscular positioning during epidermal maturation
Source: iScience. 2024 Dec 12;28(1):111577. doi: 10.1016/j.isci.2024.111577 (PMC11732705; doi:10.1016/j.isci.2024.111577)
Supplement: Document S1. Figures S1–S5 [file mmc1.pdf]

iScience, Volume *28*

Supplemental information

The BEN domain protein LIN-14 coordinates neuromuscular positioning during epidermal maturation

Eugene Jennifer Jin, Yingchuan Billy Qi, Andrew D. Chisholm, and Yishi Jin

Figure S1

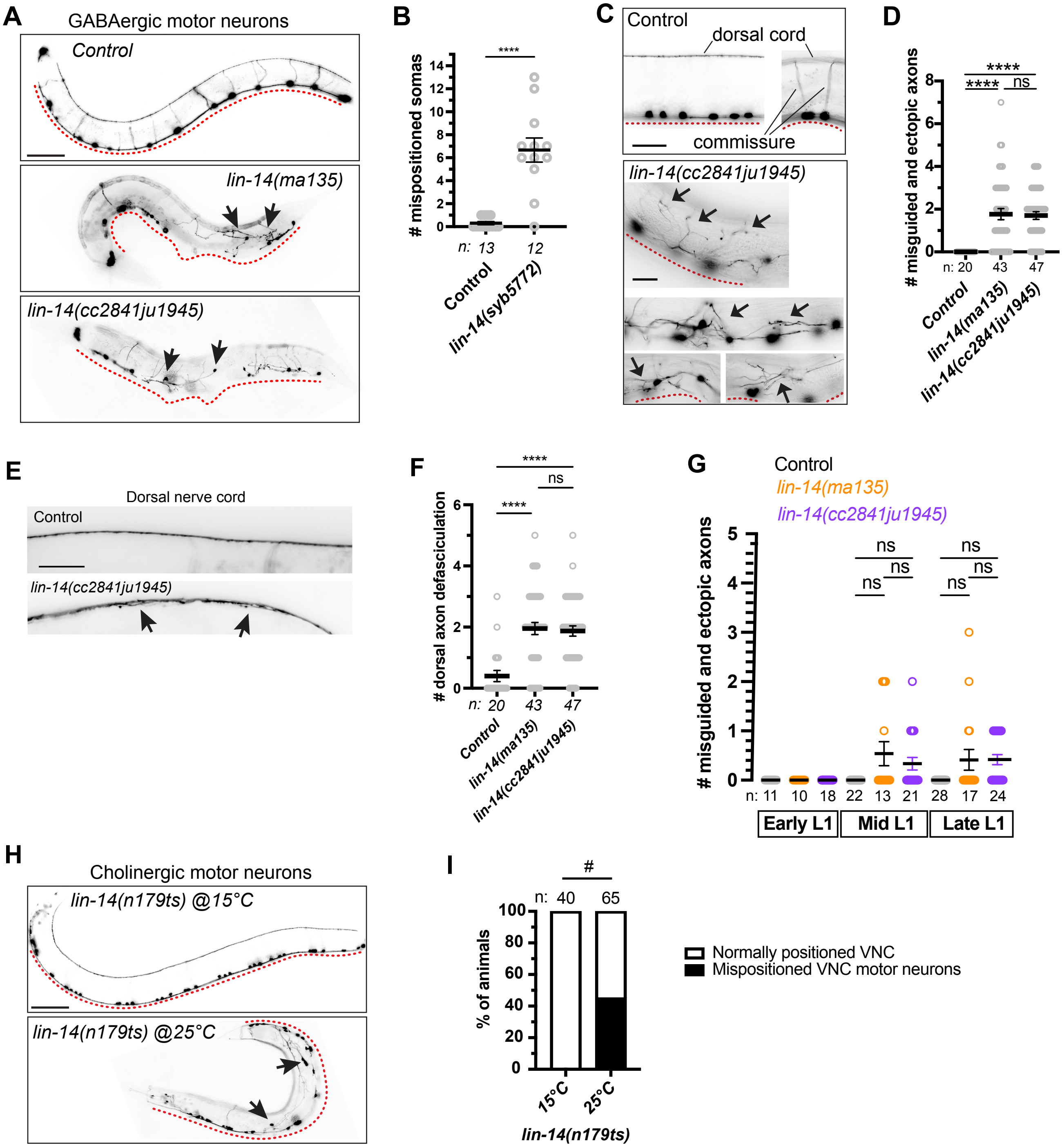

Supplemental Figure S1. Motor neuron defects in *lin-14* mutants

(A, C, E, H) Compound microscope images of GABAergic motor neurons (*juIs76[Punc-25::gfp]*) (A) and cholinergic motor neurons (*juIs14[Pacr-2::gfp]*) (C, E, H) in YA animals in control and *lin-14* mutants. Arrows: mispositioned somas (A, D), misguided and ectopic neurites (C), defasciculated DNC axons (E). Red dotted line: ventral midline. Scale: 50 µm (A, H), 20 µm (C, E). (B) Quantification of number of mispositioned cholinergic motor neuron somas, mean ± SEM. Unpaired t-test (**** $p<0.0001$). (D, G) Quantification of cholinergic motor neuron axons with misguided and ectopic neurites observed per L4 (D) or early-late L1 (G) animal. Mean ± SEM. One way ANOVA, Tukey's multiple comparisons test (D), and One way ANOVA, Šídák's multiple comparisons test (G) (ns $p>0.05$, **** $p<0.0001$). (F) Quantification of dorsal axon defasciculation in L4-YA animals in control and lin-14 mutants. Mean ± SEM. One way ANOVA, Tukey's multiple comparisons test (ns $p>0.05$, **** $p<0.0001$). (I) Quantification of mispositioned VNC cholinergic motor neurons in *lin-14(n179ts)* mutant cultured at 15°C or 25°C. Chi-squared test, Marascuilo procedure (# $p<0.05$, ns $p>0.05$).

Figure S2

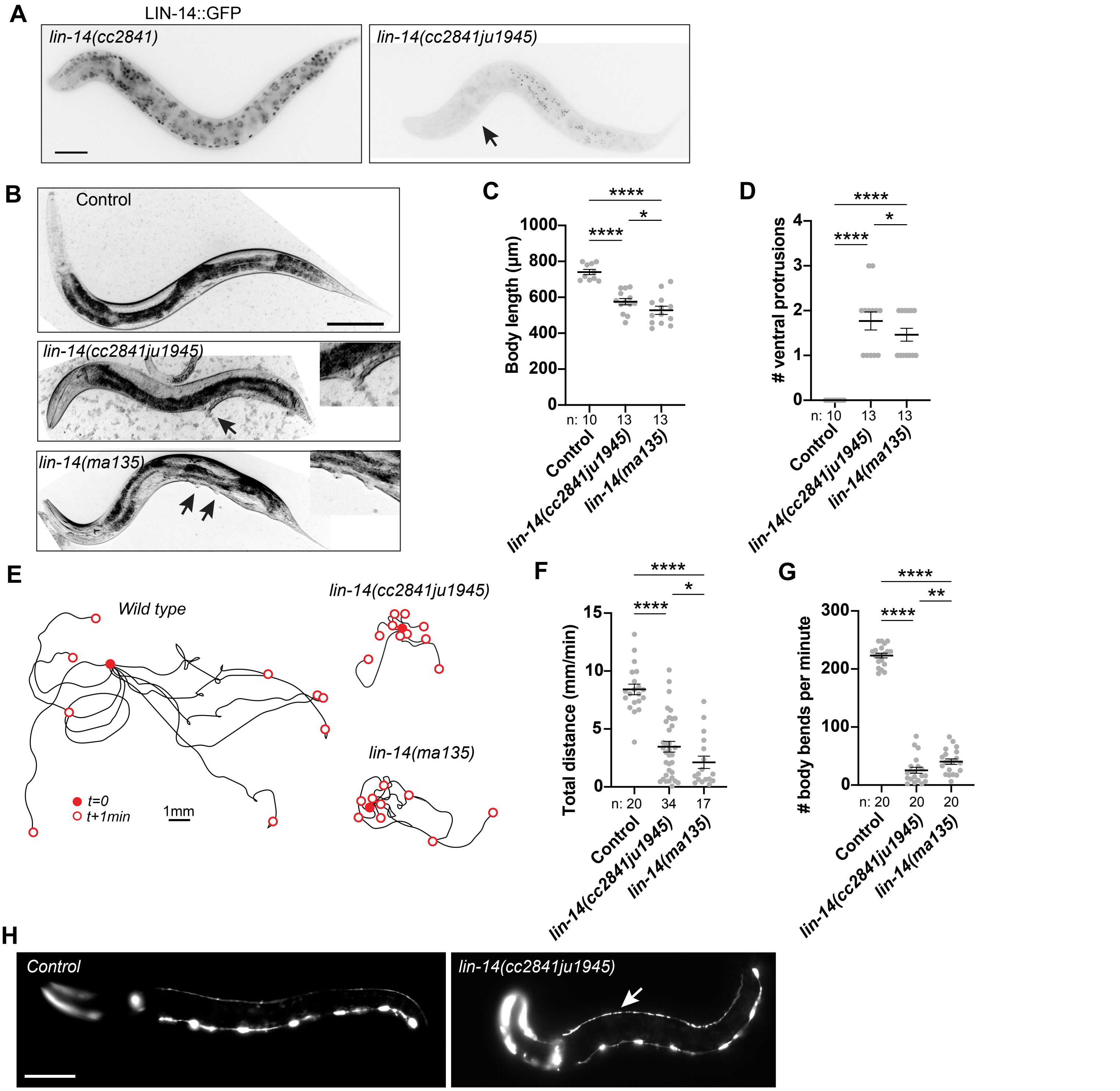

Supplemental Figure S2. ***lin-14(cc2841ju1945)*** **displays strong loss of function phenotypes of** ***lin-14***
(A) LIN-14::GFP expression in early L1 *lin-14(cc2841)* and *lin-14(cc2841ju1945)* mutant. Arrow: LIN-14::GFP under detection level. Scale: 20 µm. (B) Bright field images of L4 animals in genotypes indicated. Arrows: ventral protrusions. Scale: 100 µm. (C-D) Quantification of body length (C) and ventral protrusions (D) in L4 control and *lin-14* mutants indicated by mean ± SEM. One way ANOVA, Tukey's multiple comparisons test (* p<0.05, **** p<0.0001). (E) Locomotion traces of control and *lin-14* mutant L4s on agar plate. Solid red circle: the origin. Empty red circles: worm position after 1 minute. Black lines: traces of worm movement. Scale: 1 mm. (F-G) Quantification of total distance travelled in 1 minute (F) and thrashing rate (G) in L4 control and *lin-14* mutants. Mean ± SEM. One way ANOVA, Tukey's multiple comparisons test (* p<0.05, **p<0.01, **** p<0.0001). (H) Compound microscopy image of GABAergic presynaptic reporter *juIs236[Punc-25::wCherry::RAB-3]* in control and *lin-14* mutant early L1 animals. Arrow: precocious synapse remodeling of DD neurons. Scale: 20 µm.

Figure S3

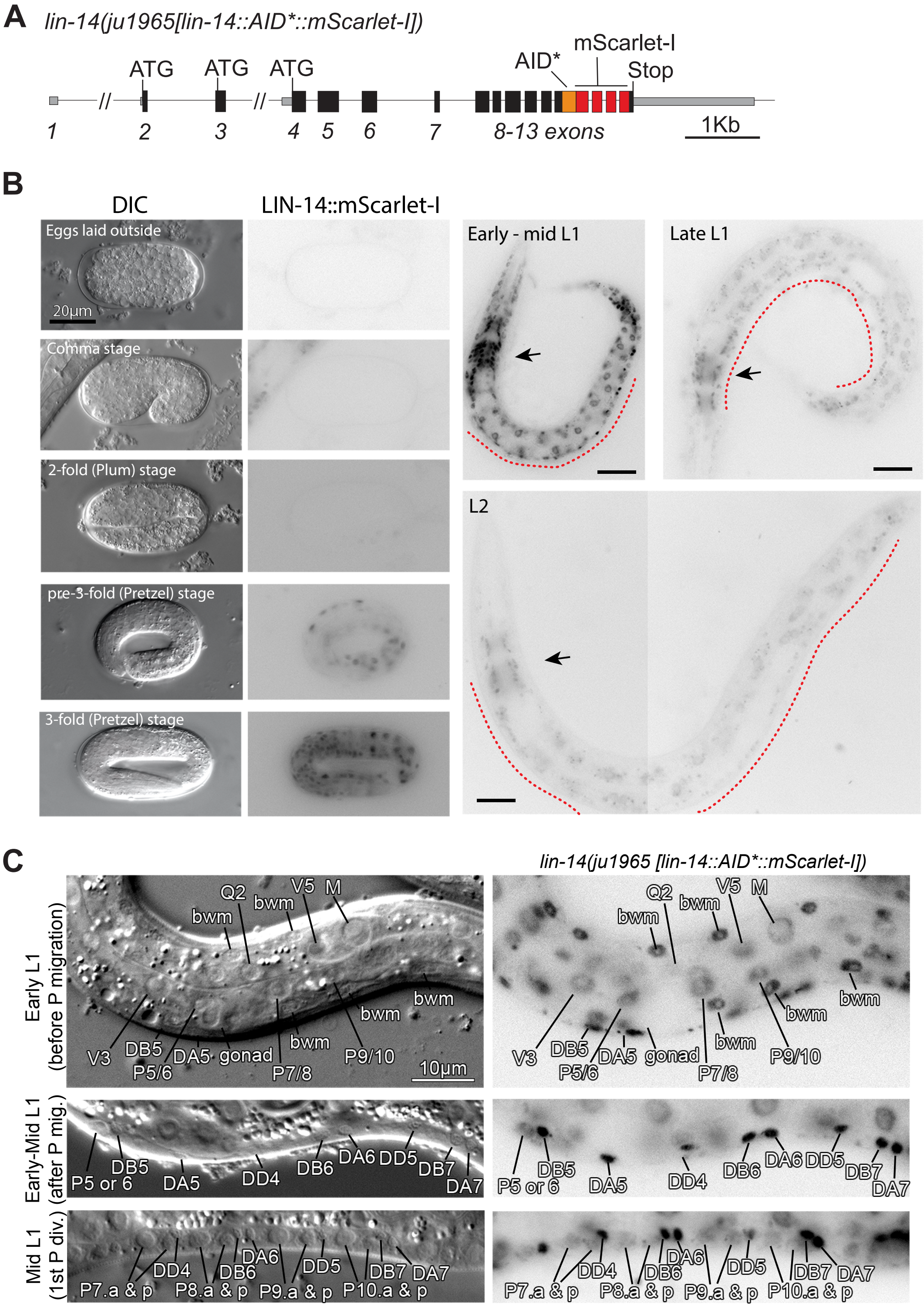

Supplemental Figure S3. LIN-14::AID*::mScarlet-I knock-in resembles endogenous LIN-14 expression
(A) Illustration of *lin-14(ju1965)*, showing AID*::mScarlet-I knock-in at 3' end of endogenous *lin-14*. (B-C) Compound microscope images of LIN-14::mScarlet-I expression in indicated embryo and larval stages. (B) Similar to the LIN-14::GFP expression, LIN-14::mScarlet-I expression begins in the three-fold stage of late embryogenesis. Expression intensity peaks in late embryos and early L1s and is then downregulated in most cells in late L1. Weak expression remains in neurons in the anterior ganglia in L2 (arrow). Red dotted line: ventral midline. Scale: 20 μm. (C) Higher resolution of LIN-14::mScarlet-I expression in nuclei of P cells and daughters (Pn.a/p), V cells, Q cells, M cell, body wall muscles (bwm), and motor neurons (DA, DB, DD) in early and mid L1s, focusing on posterior of gonad. DIC was used to identify the labelled cells based on position and shape of the nuclei. Scale: 10 μm.

Figure S4

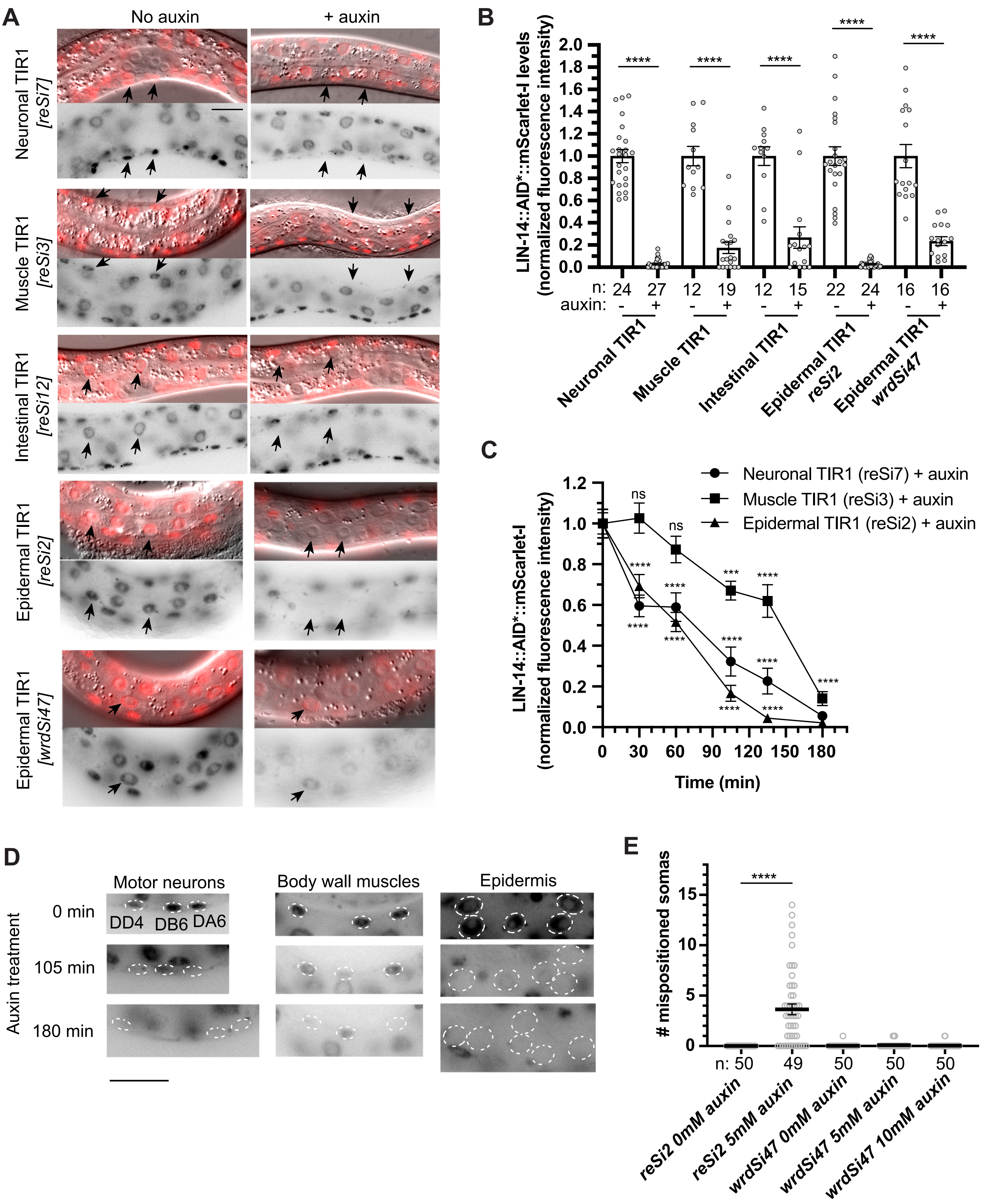

A
No auxin
+ auxin
Neuronal TIR1 [reSi7]
Muscle TIR1 [reSi3]
Intestinal TIR1 [reSi12]
Epidermal TIR1 [reSi2]
Epidermal TIR1 [wrdSi47]
B
LIN-14::AID*::mScarlet-I levels (normalized fluorescence intensity)
****
n: 24 27 12 19 12 15 22 24 16 16
auxin: - + - + - + - + - +
Neuronal TIR1
Muscle TIR1
Intestinal TIR1
Epidermal TIR1 reSi2
Epidermal TIR1 wrdSi47
C
Neuronal TIR1 (reSi7) + auxin
Muscle TIR1 (reSi3) + auxin
Epidermal TIR1 (reSi2) + auxin
LIN-14::AID*::mScarlet-I (normalized fluorescence intensity)
ns
***
****
Time (min)
D
Motor neurons
Body wall muscles
Epidermis
0 min
105 min
180 min
Auxin treatment
DD4
DB6
DA6
E
mispositioned somas
****
n: 50 49 50 50 50
reSi2 0mM auxin
reSi2 5mM auxin
wrdSi47 0mM auxin
wrdSi47 5mM auxin
wrdSi47 10mM auxin

Supplemental Figure S4. Tissue specific depletion of LIN-14::AID*::mScarlet-I

(A-B) DIC and fluorescence images (A) and quantification (B) showing LIN-14::mScarlet-I expression in nuclei (arrows) of motor neurons, body wall muscles, intestine, and lateral epidermis in control and auxin treated early L1 animals. Scale: 10 µm. Background subtracted mean fluorescence intensity in auxin treated condition was normalized to no-auxin controls. 3-4 nuclei were quantified per individual, and each datapoint represents a nucleus expression. Mean ± SEM. One way ANOVA, Šídák's multiple comparisons test (**** p<0.0001). (C-D) LIN-14::AID*::mScarlet-I degradation kinetics in motor neurons, body wall muscles, and lateral epidermis. (C) Background subtracted mean fluorescence intensity was normalized to the no-auxin controls of the same time point. 3-4 nuclei were quantified per individual animal, and 6-12 animals were quantified per condition. All data points from 2 independent repeats were pooled together. Mean ± SEM. Statistical tests were performed comparing auxin-treated vs no-auxin control per time point: One way ANOVA, Šídák's multiple comparisons test (ns p>0.05, *** p<0.001, **** p<0.0001). (D) Compound microscope images of LIN-14::AID*::mScarlet-I expression in motor neurons (DD4, DB6, DA6), dorsal body wall muscles, and lateral epidermis over time course of 5 mM auxin treatment. Scale: 10 µm. (E) Quantification of mispositioned cholinergic motor neuron somas in L4. Mean ± SEM. Unpaired t test (**** p<0.0001).

Figure S5

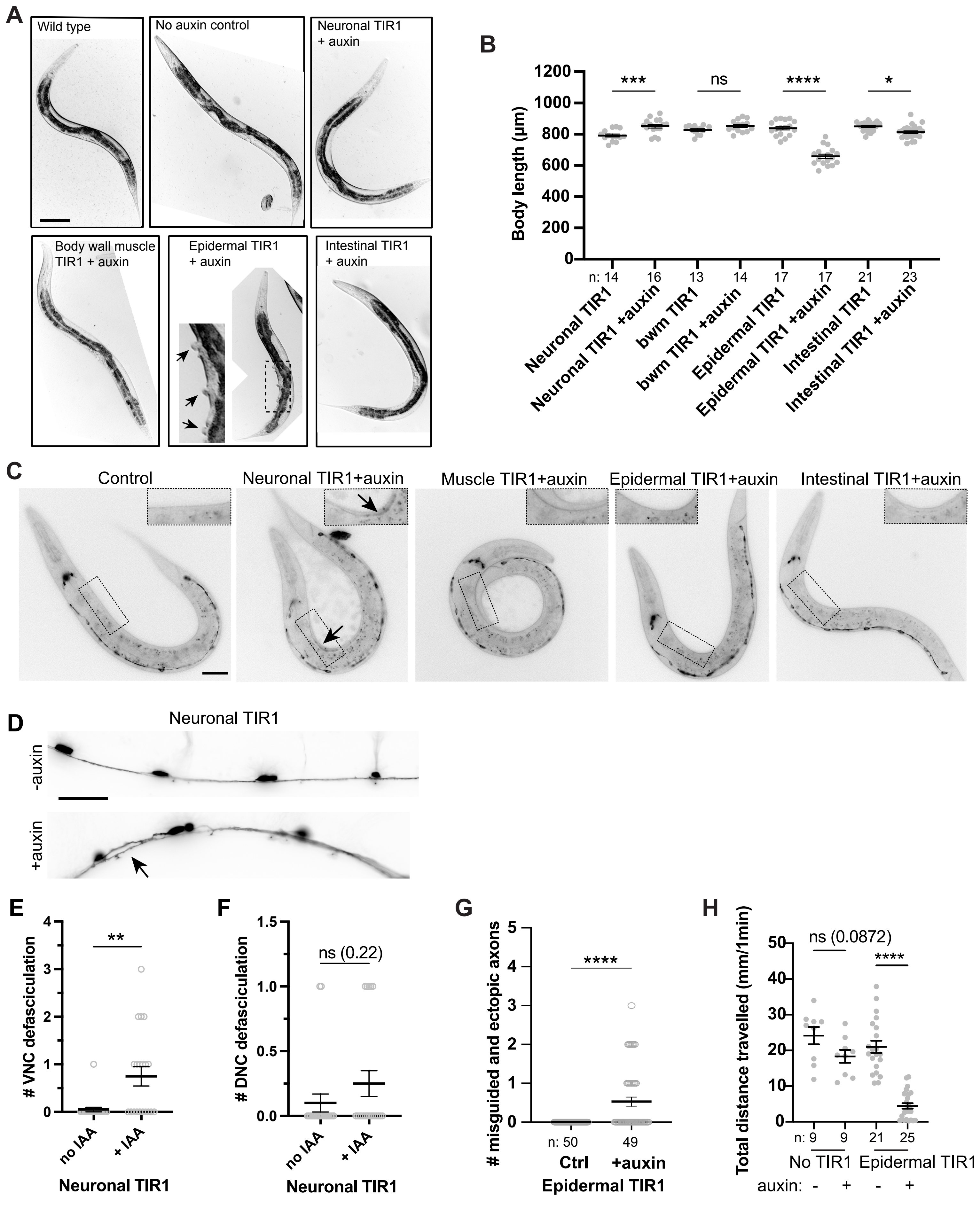

A
Wild type
No auxin control
Neuronal TIR1 + auxin
Body wall muscle TIR1 + auxin
Epidermal TIR1 + auxin
Intestinal TIR1 + auxin
B
Body length (μm)
***
ns
****
*
n: 14 16 13 14 17 17 21 23
Neuronal TIR1
Neuronal TIR1 +auxin
bwm TIR1
bwm TIR1 +auxin
Epidermal TIR1
Epidermal TIR1 +auxin
Intestinal TIR1
Intestinal TIR1 +auxin
C
Control
Neuronal TIR1+auxin
Muscle TIR1+auxin
Epidermal TIR1+auxin
Intestinal TIR1+auxin
D
Neuronal TIR1
-auxin
+auxin
E
VNC defasciculation
**
no IAA
+ IAA
Neuronal TIR1
F
DNC defasciculation
ns (0.22)
no IAA
+ IAA
Neuronal TIR1
G
misguided and ectopic axons
****
n: 50 49
Ctrl
+auxin
Epidermal TIR1
H
Total distance travelled (mm/1min)
ns (0.0872)
****
n: 9 9 21 25
No TIR1
Epidermal TIR1
auxin: - + - +

Supplemental Figure S5. Epidermal depletion of LIN-14::AID*::mScarlet-I causes *lin-14* mutant phenotypes
(A) Brightfield images showing gross morphology of L4 animals after tissue specific depletion of LIN-14. Arrows: ventral protrusions. Scale: 100 µm. (B) Quantification of body length of L4 animals expressing tissue specific TIR1 transgenes with or without auxin treatment. Mean ± SEM. One way ANOVA, Šídák's multiple comparisons test (ns p>0.05, * p<0.05, *** p< 0.001, **** p<0.0001). (C) Compound microscope images showing *juIs1[Punc-25::SNB-1::GFP]* expression in mid L1 animals after LIN-14 depletion in neurons, body wall muscles, epidermis or intestine. Arrow: precocious DD synapse remodeling. Scale: 20 µm. (D) Compound microscope images showing VNC cholinergic motor neurons (*juIs14[Pacr-2::gfp]*) in L4 animals. Arrow: axon defasciculation shown by two distinct VNC axons. Scale: 20 µm. (E-G) Quantification of VNC defasciculation (E), DNC defasciculation (F), and misguided and ectopic axons (G) in L4 animals. Mean ± SEM. Unpaired t test (ns p>0.05, ** p<0.01, **** p<0.0001). (H) Quantification of total distance travelled in 1 minute in L4 animals. Mean ± SEM. One way ANOVA, Šídák's multiple comparisons test (ns p>0.05, **** p<0.0001).
